# Biobased Compounds and the Circular Economy: A Bibliometric Review of Waste Valorization and Environmental Sustainability

**DOI:** 10.3390/molecules31183262

**Published:** 2026-09-15

**Authors:** Segundo Rojas-Flores, Anibal Alviz-Meza, Angel Dario Gonzalez-Delgado

**Affiliations:** 1 Institutos y Centro de Investigación, Universidad Cesar Vallejo, Trujillo 13001, Peru; 2 Nanomaterials and Computer Aided Process Engineering Research Group (NIPAC), Chemical Engineering Department, Universidad de Cartagena, Cartagena 130014, Bolivar, Colombia; aalvizm1@unicartagena.edu.co (A.A.-M.); agonzalezd1@unicartagena.edu.co (A.D.G.-D.)

**Keywords:** biobased compounds, sustainability, circular economy, biopolymers, agricultural waste, environmental transition

## Abstract

The global plastic crisis, driven by 367 million tons of annual production and persistent environmental contamination, has intensified research into biobased compounds as sustainable alternatives. However, significant gaps remain in understanding the scientific landscape, research priorities, and industrial scalability of these materials. This study conducts a comprehensive bibliometric and systematic review to map the knowledge structure of biobased compounds and circular economy research. A refined search strategy was executed in Scopus (June 2026), retrieving 1350 documents, which were screened following PRISMA guidelines, resulting in a final dataset of 1306 peer-reviewed documents. The search strategy was designed to be comprehensive, capturing both the core biobased compounds literature and adjacent sustainability science (e.g., life cycle assessment, circular economy frameworks) that provides essential methodological foundations for the field. Bibliometric analyses were performed using RStudio/bibliometrix for descriptive statistics, VOSviewer for co-authorship and keyword co-occurrence networks, and Plotly Studio for interactive visualizations. The results reveal exponential growth in scientific production (R^2^ = 0.92046), with publications increasing from 15 in 2010 to 95 in 2026 (partial year, data through June). India leads in publication volume (293 documents), while the United States exhibits the highest citation impact (114.74 citations per paper). “Sustainable development” (489 occurrences) and “circular economy” (419 occurrences) are the dominant keywords, with the latter showing the highest annual growth rate (2333.33%). The *Journal of Cleaner Production* (31 publications, 2040 citations) and *Bioresource Technology* (95.53 average citations) are the most productive and impactful journals, respectively. Ten transitional research gaps were identified, with “synthetic biology” and “bioaccumulation” showing the highest priority indices (122.16). The most common barriers are weak theoretical foundations and limited research duration, while mechanical properties and reprocessability face additional interdisciplinary complexity. These findings indicate that biobased compounds research is rapidly expanding, suggesting a need for strengthened theoretical frameworks and increased academic attention to interdisciplinary collaboration to achieve circular economy transitions aligned with Sustainable Development Goals 12 and 13.

## 1. Introduction

The contemporary environmental crisis is intrinsically linked to the dependence on petroleum-based plastics, whose global production reached 367 million tons in 2020, consuming between 4% and 6% of the world’s crude oil [1]. It is estimated that 7 billion tons of plastic waste is generated annually, but only 10% is recycled, resulting in up to 12.7 million tons of these materials contaminating oceans each year [2,3]. Although bioplastics can reduce greenhouse gas emissions by up to 25%, and biobased PET emits 25% less CO_2_ than its fossil counterpart, their current market share represents barely 1% of total plastic production [4]. In parallel, waste underutilization is critical: each year, 2 billion tons of lignocellulosic biomass is produced, and approximately one-third of the food produced globally is wasted [5]. Aligning with the Sustainable Development Goals (SDGs)—specifically SDG 12 (Responsible Consumption and Production), which promotes sustainable production patterns and resource efficiency, and SDG 13 (Climate Action), which calls for urgent measures to combat climate change and its impacts—these materials offer a renewable and compostable alternative that can drastically reduce the carbon footprint and valorize biomass that would otherwise go to waste [6,7]. Although the use of recycled materials such as PLA can maintain mechanical performance with recovery rates of 92.7%, the transition toward an effective circular economy is hindered by high scalability costs and the lack of standardized methods [8]. This disconnect between the massive volume of available waste and its integration into sustainable production cycles demands a bibliometric review that synthesizes trends and gaps in the valorization of biobased compounds.

To address the plastic waste problem, circular economy models integrating biopolymers such as PLA and PHA derived from lignocellulosic biomass are being adopted [9]. A fundamental pathway is the development of green polymer nanocomposites, which incorporate nanofillers to optimize mechanical properties and reduce the water sensitivity of biobased materials [10]. Similarly, integrated biorefineries employ microorganisms to ferment carbohydrates from industrial waste, transforming them into high-value chemical building blocks [2,6]. The implementation of chemical recycling via pyrolysis enables the efficient recovery of valuable monomers, closing the production cycle in a sustainable manner and significantly reducing dependence on virgin fossil-based raw materials [11]. In this context, Abu-Zurayk et al. (2025) demonstrated that PLA reinforced with 1% cellulose nanocrystals achieved a 64.17% increase in tensile modulus compared to the pure polymer [12]. Likewise, Weerarathna et al. (2025) evaluated polyurethane foams based on 100% lignin polyol, achieving a 44% increase in mechanical resistance [13]. Müller et al. (2025) analyzed demographic environmental behaviors through surveys, finding that consumption models explained sustainability gaps with an R^2^ coefficient of up to 0.64 [14]. Similarly, Mocerino (2025) investigated polymers for resilient architecture, demonstrating that long-chain branching in PLA enabled a 30% reduction in structural weight [15].

The importance of a bibliometric analysis on biobased compounds and the circular economy lies in its capacity to identify research gaps and opportunities in multi-feedstock systems [16]. The use of the Scopus database enables the extraction of high-quality metadata, which, when processed in RStudio using the Bibliometrix package, facilitate the visualization of scientific evolution and global collaboration networks in areas such as bioenergy [17]. Complementary tools such as VOSviewer enable literature mapping that refines the understanding of sustainability trends and the evolution of regenerative design concepts [18]. Furthermore, a meta-level bibliometric analysis helps assess how materials science contributes to the Sustainable Development Goals, highlighting international collaboration in biotechnology and polymer research [19]. The integration of Plotly Studio adds dynamism to statistical analysis, allowing the monitoring of exponential publication growth in critical areas such as polylactic acid and cellulose nanocomposites [20]. This systematic approach, which often follows PRISMA protocols, ensures rigorous article selection from indexed repositories to inform market dynamics and environmental safety [21]. Process Systems Engineering (PSE) benefits from such analyses by integrating data-driven models for optimal decision-making, promoting an effective transition toward the use of biobased plastics and efficient end-of-life management [22]. This research aims to address the disconnect between the large volume of available residual biomass and its limited industrial integration, providing a scientific-metric framework to guide valorization toward sustainable circular production cycles.

The primary objective of this study is to conduct a comprehensive knowledge mapping and identify emerging trends through the integration of scientometric and systematic analyses on the role of biobased compounds in the environmental transition. This methodology enables the structuring of the literature corpus to detect research gaps and propose a roadmap toward an effective circular bioeconomy. The study is organized around five research questions: Q1 analyzes temporal publication and citation trends to assess scientific growth, which has shown an exponential increase since 2018 in areas such as biopolymers and PHA. Q2 seeks to determine the spatial distribution and geographical collaboration networks among leading institutions and countries in biobased research, including India, the United States, and European nations. Q3 focuses on identifying dominant themes, such as green nanocomposites and integrated biorefineries, through keyword co-occurrence analysis. Q4 aims to delineate predominant thematic areas and the highest-impact journals leading the dissemination of these findings. Finally, Q5 evaluates product properties, processing techniques (such as extrusion and compression molding), and critical industrial scalability challenges. Additionally, key biomass sources (such as Opuntia, algae, agricultural waste, and fish collagen) are compared, along with their direct linkage to SDGs 12 and 13, to identify emerging trends that may inform future research agendas toward waste valorization and effective environmental sustainability.

## 2. Results and Discussion

**Figure 1:**
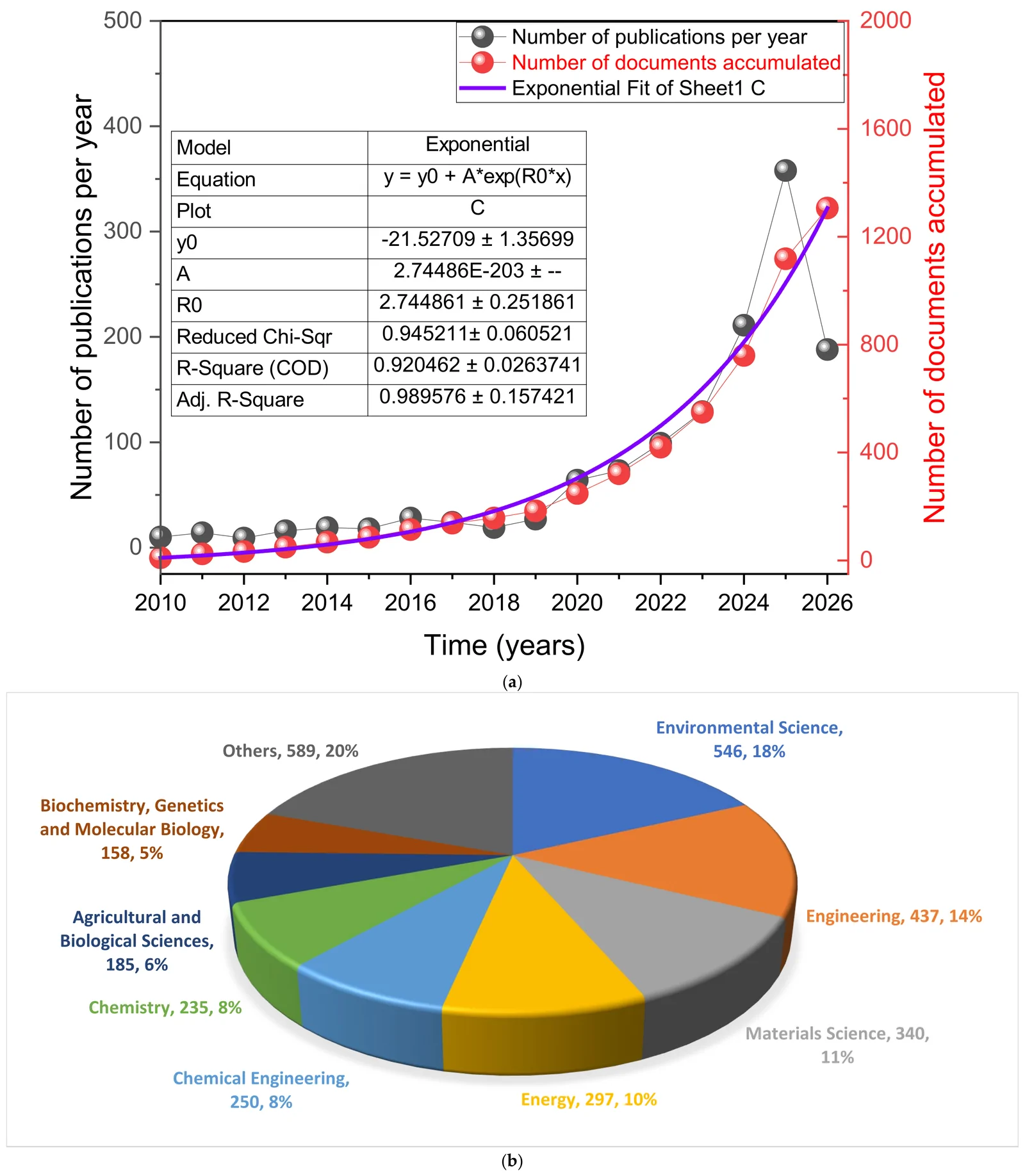
(**a**) Temporal evolution of scientific production on biobased compounds and circular economy (2010–2026), showing annual publications and cumulative publications. The exponential growth trend is confirmed by an R^2^ value of 0.9654, with annual growth exceeding 8% in recent years. (**b**) Relative distribution of publications by thematic area.

During the period considered (2010–2026, with 2026 data representing a partial year through June), it can be observed that until 2019, the cumulative number of publications on the topic increased modestly, with a nearly constant number of documents per year (Figure 1a). From 2020 onwards, the number of publications per year has steadily increased, with a particularly sharp rise between 2024 and 2025. Consequently, the cumulative number of publications is growing exponentially, with an R^2^ value of 0.92046 confirming the accelerated growth trend. This behavior reflects the growing interest of the scientific community in sustainable alternatives to petroleum-derived plastics, driven by the urgency of addressing the plastic pollution crisis and the transition toward circular production models [23]. The constant increase in publication volume suggests a maturation of knowledge, but also poses challenges in terms of thematic saturation and the need to diversify into less explored niches [24,25]. Figure 1b, in turn, breaks down the relative distribution of publications by thematic area, offering a complementary perspective on the disciplinary foci that dominate this field of study. The areas of Agricultural and Biological Sciences, Biochemistry, Genetics and Molecular Biology, and environmental science concentrate the highest proportion of documents, reflecting the multidisciplinary nature of research on biobased compounds [26]. This preeminence of biological and environmental sciences is consistent with the focus on the extraction of bioactive compounds, the characterization of biopolymers, and the assessment of ecological impacts—processes that constitute the scientific foundation for the design of sustainable materials [27]. Likewise, the significant presence of areas such as chemical engineering and materials science indicates a growing interest in industrial scalability and the optimization of the mechanical and thermal properties of bioproducts, aspects that are critical for their competitiveness against conventional plastics [28]. Notably, the inclusion of areas such as Pharmacology, Toxicology, and Pharmaceutics evidences the exploration of biomedical and pharmaceutical applications, which broadens the spectrum of biomass valorization opportunities. Overall, the thematic distribution reveals an integrated scientific ecosystem where fundamentally basic approaches converge with technological applications and sustainability assessments, thus establishing a bridge between fundamental knowledge and its translation into tangible solutions for industry. This thematic mapping constitutes a valuable tool for identifying interdisciplinary synergies and guiding future R&D investments toward areas with greater potential for environmental and economic impact.

The ten most cited documents presented in Table 1 reveal the bibliometric landscape of biobased products and sustainable materials. Published in prestigious journals such as *Nature*, *Science*, and *Nature Reviews Materials*, they offer a compelling window into the thematic priorities, methodological approaches, and conceptual frameworks that have driven research on the transition toward a circular bioeconomy. At the apex of this corpus is the review by Zhu et al. (2016) on sustainable polymers from renewable resources [29], which has accumulated an impressive 2502 total citations. This foundational work established the technical and economic case for replacing petrochemical-based plastics with biobased alternatives, addressing critical issues regarding feedstock availability, polymer synthesis, and end-of-life scenarios. Building on this foundation, the more recent review by Rosenboom et al. (2022) on bioplastics for a circular economy [30] has already garnered 1847 citations, with an annual rate of 461.75—the highest among all documents in the table. This remarkable acceleration in scholarly attention signals a paradigm shift: whereas early research focused primarily on demonstrating the feasibility of bioplastics, contemporary scholarship increasingly interrogates their integration into circular economic frameworks, considering aspects such as collection, sorting, mechanical recycling, and chemical upcycling.

The research by Li et al. (2021) on developing fibrillated cellulose as a sustainable technological material [31], with 1513 citations and an annual rate of 302.6, exemplifies the growing interest in high-performance biobased materials that transcend conventional packaging applications. Cellulose nanofibers offer exceptional mechanical properties, renewability, and functional versatility, positioning them as viable candidates for applications ranging from composites and coatings to biomedical devices and energy storage. This trajectory toward advanced functionality is complemented by the review on polylactic acid (PLA) by Castro-Aguirre et al. (2016) [32], which has accumulated 1222 citations. PLA serves as a paradigmatic case study of the challenges in biopolymer commercialization: although it is one of the most widely produced bioplastics, its performance limitations, processing requirements, and end-of-life options continue to inspire extensive research into blend formulations, nanocomposite reinforcement, and chemical recycling strategies.

**Table 1:** Top 10 most cited documents on biobased products, sustainable materials, and circular economy.

	Title	Total Citations	Citations per Year	Publication Year	Journal	First Author	Document Type
1	Sustainable polymers from renewable resources [29].	2502	250.2	2016	*Nature*	Zhu Y.	Review
2	Bioplastics for a circular economy [30].	1847	461.75	2022	*Nature Reviews Materials*	Rosenboom J.-G.	Review
3	Developing fibrillated cellulose as a sustainable technological material [31].	1513	302.6	2021	*Nature*	Li T.	Review
4	Poly(lactic acid)—Mass production, processing, industrial applications, and end of life [32].	1222	122.2	2016	*Advanced Drug Delivery Reviews*	Castro-Aguirre E.	Review
5	Toward sustainable and systematic recycling of spent rechargeable batteries [33].	977	122.12	2018	*Chemical Society Reviews*	Zhang X.	Review
6	Composites from renewable and sustainable resources: Challenges and innovations [34].	961	120.12	2018	*Science*	Mohanty A.K.	Review
7	How circular is the global economy?: An assessment of material flows, waste production, and recycling in the European union and the world in 2005 [35].	897	81.55	2015	*Journal of Industrial Ecology*	Haas W.	Article
8	Sustainability of biodegradable plastics: New problem or solution to solve the global plastic pollution? [36].	723	180.75	2022	*Current Research in Green and Sustainable Chemistry*	Moshood T.D.	Review
9	Environmental performance of bio-based and biodegradable plastics: The road ahead [37].	722	80.22	2017	*Chemical Society Reviews*	Lambert S.	Review
10	The ten principles of green sample preparation [38].	640	160	2022	*TrAC—Trends in Analytical Chemistry*	Lopez-Lorente A.I.	Review

Note: The table includes publications that extend beyond the core topic of biobased compounds (e.g., battery recycling, green sample preparation, sustainable concrete) because they provide foundational methodological frameworks—such as life cycle assessment, circular economy indicators, and green chemistry principles—that are directly applicable to biobased materials research. Their inclusion reflects the interdisciplinary nature of the field and the comprehensive scope of the search strategy.

The inclusion of Haas et al. (2015) on the circularity of the global economy [35], with 897 citations, is particularly notable as this is the sole article (rather than review) in this top ten list. This work provided one of the first quantitative assessments of material flows, waste production, and recycling rates at the global and European scales, establishing empirical benchmarks that have informed subsequent policy debates and research agendas. Its presence among the most cited documents underscores the critical importance of systems-level analysis in validating the environmental claims of biobased alternatives. Similarly, the review by Lambert and Wagner (2017) on the environmental performance of biobased and biodegradable plastics [37], with 722 citations, directly confronts the contested question of whether bioplastics offer genuine environmental benefits or merely shift burdens from one impact category to another. This critical perspective has become increasingly salient as life cycle assessment methodologies have matured and evidence has accumulated regarding the greenhouse gas emissions, land-use change, and ecotoxicity associated with certain biobased feedstocks.

More recent publications reflect an expansion of scope beyond conventional plastics to encompass broader sustainability challenges. The review by Zhang et al. (2018) on the sustainable and systematic recycling of spent rechargeable batteries [33], with 977 citations, illustrates the convergence of circular economy principles with the critical materials demands of the energy transition. The recovery of lithium, cobalt, and rare earth elements from end-of-life batteries addresses both resource security and waste management concerns while mitigating the environmental and social impacts of primary mining. This thematic expansion is further evidenced by the work of Mohanty et al. (2018) on composites from renewable and sustainable resources [34], with 961 citations, which extends the circular economy framework to high-performance structural materials. The integration of natural fibers, biobased resins, and sustainable manufacturing processes offers pathways to reduce the carbon footprint of the automotive, construction, and consumer goods sectors. The review by López-Lorente et al. (2022) on the ten principles of green sample preparation [38], with 640 citations, introduces a methodological dimension often overlooked in discussions of sustainable materials. The development of analytical techniques that minimize solvent consumption, reduce energy use, and generate less hazardous waste is essential for ensuring that the entire value chain—from feedstock characterization to product quality control—aligns with sustainability principles. Finally, the work by Moshood et al. (2022) on the sustainability of biodegradable plastics [36], with 723 citations and an annual rate of 180.75, encapsulates the field’s growing self-awareness: biodegradable plastics are not an unconditional solution but rather a complex intervention that must be carefully designed, implemented, and managed to avoid unintended consequences such as microplastic formation, methane emissions from anaerobic degradation, and contamination of conventional recycling streams.

Collectively, these ten publications reveal a research domain that has matured from exploratory demonstrations of biobased feasibility to sophisticated analyses of systemic integration, life cycle performance, and circular economy alignment. The predominance of review articles (nine out of ten) suggests that the field remains in a phase of knowledge consolidation, synthesizing dispersed empirical findings into coherent frameworks. However, the growing attention to specific applications—batteries, composites, and analytical chemistry—and the increasing emphasis on critical assessment rather than advocacy indicate a trajectory toward greater methodological rigor and practical relevance.

It is noteworthy that the top-cited documents include publications not exclusively focused on biobased compounds, such as the review on battery recycling, the principles of green sample preparation, and the life cycle assessment of green concrete mixes. This thematic diversity reflects the broader intellectual ecosystem in which biobased compounds research is embedded. These works provide essential methodological and conceptual tools—including life cycle assessment frameworks, circular economy metrics, and green chemistry principles—that are directly applicable to the evaluation and development of biobased materials. Their presence among the most cited documents underscores the importance of interdisciplinary knowledge transfer and validates the comprehensive search strategy employed in this study, which aimed to capture not only the core biobased compounds literature but also the adjacent sustainability science that informs it.

Table 2 presents an analysis of global scientific leadership in research on biobased products and sustainable materials. This analysis must be interpreted with caution, as bibliometric indicators reflect publication patterns and academic visibility but should not be extrapolated as direct measures of scientific leadership, technological maturity, or industrial innovation capacity, as has been noted in recent methodological studies [17,18]. India leads scientific output with 293 publications and 4211 total citations. This high publication volume is consistent with the country’s substantial research capacity and its status as a major producer of agricultural biomass, but bibliometric indicators alone cannot confirm causal relationships between biomass availability and research output [1,2]. The H-index of 33 confirms a consistent and broad-spectrum presence in the field. However, the average citations per publication (14.37) is moderate compared to other nations, which may indicate that, despite the high volume, the individual impact of documents is more limited. This pattern is consistent with the observation that production in countries with abundant biomass resources tends to be quantitatively significant but qualitatively heterogeneous [3,4]. Italy, with 103 publications, stands out for its high average citations per document (35.68) and an H-index of 30, demonstrating high-quality research with international visibility. Institutions such as the Smart Materials group at the Italian Institute of Technology lead this production, focusing on the development of nanocomposites and functional biopolymers [9,10]. China, with 102 publications, presents the highest average citations among Asian countries (37.7) and an H-index of 25. The thematic analysis of Chinese publications reveals a concentration on bioplastic production from lignocellulosic waste and biorefinery process optimization [5,6], which may suggest a strategic orientation toward high-impact applications, though this interpretation requires confirmation through policy and funding analyses.

The United States, although ranking fourth in volume with 58 publications, exhibits the highest average citation rate (114.74). This pattern is consistent with publication in high-impact-factor journals and with interdisciplinary approaches that connect materials science with circular economy and life cycle assessment [7,8]. However, citation impact reflects academic visibility, not a direct measure of scientific influence or technological leadership. Germany and the United Kingdom show similar patterns, with average citations of 62.15 and 102.97 respectively, reinforcing the idea that quality and academic visibility are not necessarily correlated with production volume [11,12]. Spain and Malaysia occupy intermediate positions, with 48 and 45 publications respectively, highlighting their focus on agro-industrial waste valorization and biopolymer production from renewable sources [13,14]. Brazil and Poland, despite lower volumes, have publications concentrated in bioplastic applications for packaging and construction sectors [15,16], which aligns with the Sustainable Development Goals. However, publication volume does not directly measure industrial interest or policy alignment. The results in Table 2 reveal a diverse scientific landscape where volumetric production does not equate to academic impact. India leads in quantity, while nations such as the United States, Germany, and the United Kingdom excel in visibility. This distribution highlights the diverse research landscape, with some countries contributing high volume and others high impact. These bibliometric patterns indicate that the transition toward a circular bioeconomy may benefit from both intensive research in biomass-rich countries and methodological rigor in impact-oriented nations, though this interpretation remains a hypothesis requiring further investigation through policy and technology transfer analyses [19,20,21,22]. Furthermore, the absence of African and Latin American countries among the top rankings indicates a geographical gap that should be addressed through scientific cooperation and technology transfer policies [23,24,25]. Future studies should complement these indicators with patent and innovation project analyses to obtain a more complete picture of actual technological leadership [26,27,28].

**Figure 2:**
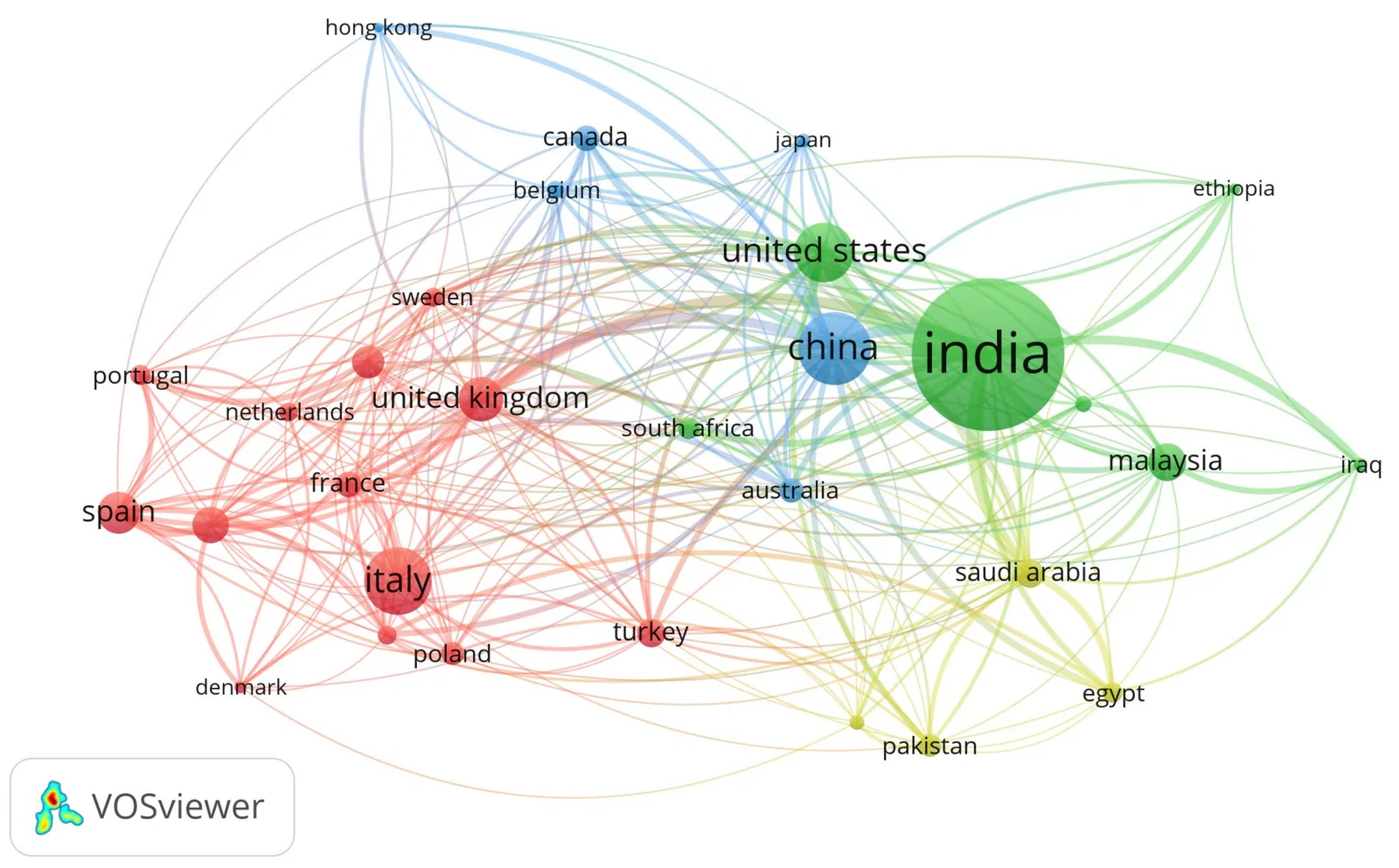
Country co-authorship network (or collaboration map).

Figure 2 presents the co-authorship network map among countries researching biobased compounds, sustainable materials, and the circular economy. This visualization should be interpreted as a reflection of structural patterns of scientific collaboration, not as a hierarchical measure of leadership or national technological capabilities [17,18]. Co-authorship network analysis enables the identification of interaction patterns and knowledge flows but should not be extrapolated to infer scientific superiority or industrial innovation capacity [19,20]. The map reveals a structure organized into four main clusters, each representing groups of countries with strong collaborative links. The most prominent cluster, shown in yellow, is dominated by India, which acts as a central node directly connected to Turkey. India, with 293 publications and 4211 total citations (Table 2), demonstrates notable research activity, supported by its vast scientific infrastructure and its position as one of the world’s largest producers of agricultural biomass [1,2]. The connection with Turkey suggests knowledge exchange in areas such as agro-industrial waste valorization and biopolymer production from renewable sources [3,4]. However, centrality in the network should not be automatically interpreted as scientific leadership, but rather as an indicator of high productivity and active collaboration [21,22].

The red cluster groups European and Asian powers such as Italy, Spain, the United Kingdom, and South Korea. Italy, with 103 publications and an average citation rate of 35.68, stands out for its high-quality research in nanocomposites and functional biopolymers [9,10]. Spain, with 48 publications and an average of 39.27, positions itself as a key player in agro-industrial waste valorization and bioplastic production [13,14]. This cluster reflects consolidated collaboration within the European space, which has been fundamental for advancing life cycle assessment and biorefinery process optimization [7,8]. The connection with South Korea suggests growing Asian interest in the circular economy, aligned with the Sustainable Development Goals [23,24]. The blue cluster integrates the United States, China, Iran, and Canada. The United States, with 58 publications and the highest average citation rate (114.74), demonstrates a disproportionately high academic influence, attributable to publication in high-impact-factor journals and the integration of interdisciplinary approaches connecting materials science with sustainability [5,6]. China, with 102 publications and an average of 37.7, reflects a research strategy oriented toward high-impact applications such as bioplastic production from lignocellulosic waste [11,12]. The connections between China, Iran, and the United States indicate dynamic knowledge exchange in areas such as chemical engineering and bioproducts, although collaboration with the United States may be particularly relevant for the transfer of advanced methodologies [25,26]. The green cluster, which features India, Malaysia, and the United States as its main contributors, demonstrates emerging research capacity in biobased compounds and growing integration into the global research network, albeit with generally lower overall citation volumes [27,28].

Figure 2 reveals that scientific collaboration on biobased compounds spans multiple geographic regions, with co-authorship links between countries that have high biomass availability (e.g., India, Malaysia) and those with established research infrastructure (e.g., the United States, Germany, the United Kingdom). This pattern is consistent with knowledge exchange, but bibliometric networks do not provide direct evidence of technology transfer or the quality of collaboration. However, the absence of African and Latin American countries in the network suggests a geographical gap that should be addressed through scientific cooperation and technology transfer policies [29,30,31]. Future studies should complement this analysis with patent and innovation project indicators to obtain a more complete picture of actual technological leadership [32,33]. Furthermore, the network structure reflects historical collaboration patterns that may not be static; changes in scientific policies and research priorities could alter these configurations in the future [34,35,36,37,38].

Table 3 presents a comprehensive bibliometric snapshot of the leading journals shaping research on biobased compounds and the circular economy, providing critical data on publication output, citation impact, and thematic influence. This ranking not only quantifies productivity but also illuminates the intellectual hubs where the most transformative ideas are being forged. At the forefront of sheer volume stands the *Journal of Cleaner Production*, with 31 publications and a commanding total of 2040 citations, averaging 65.81 citations per paper. Its dominant position is reinforced by the most cited work within this journal—Gursel et al. [39] on the life cycle assessment of green concrete mixes incorporating rice husk ash, which has accumulated 226 citations. Beyond these top-tier performers, a diverse range of specialized journals address specific niches within the broader biobased economy, each contributing unique perspectives and critical advances. *Sustainability (Switzerland)*, with 27 publications and 810 total citations, features the highly influential review by Clauser et al. [40] on the valorization of biomass waste as a sustainable raw material for energy and fuels. With 285 citations, this paper is among the most referenced in the entire dataset, highlighting the growing imperative to transform agricultural and industrial residues into valuable energy vectors. *Polymers* and the *International Journal of Biological Macromolecules* each published 23 papers, though they exhibit contrasting impact profiles. The former has accumulated 967 citations, averaging 42.04 per publication, with the review by Di Bartolo et al. [41] on bioplastics and their integration into the circular economy leading at 104 citations. The latter, while having a lower total (493 citations and an average of 21.43), anchors its influence on the comprehensive review by Pei et al. [42] on starch-based sustainable edible films loaded with bioactive components, which has received 134 citations and represents a significant breakthrough in active and intelligent food packaging solutions. The intersection of materials recycling and structural engineering is robustly covered by *Resources, Conservation and Recycling*, which has 22 publications and 1103 total citations, and published the pivotal work by Xu et al. [43]. This systematic review on factors affecting the properties of thermally activated recycled cement has been cited 243 times, linking construction waste management directly with resource efficiency and low-carbon building practices.

Complementing this engineering perspective, *Science of the Total Environment* delivers the single most cited paper in the entire table—the comprehensive framework by Elsacker et al. [44] for producing mycelium-based lignocellulosic composites, which has amassed an impressive 344 citations. With 17 publications and 985 total citations, averaging 57.94 per paper, this journal demonstrates that biodegradable structural alternatives are a rapidly maturing research frontier, capturing the attention of both environmental scientists and materials engineers. This underscores the journal’s pivotal role in bridging sustainable materials science with industrial ecology, particularly in the construction sector. In terms of average academic impact, however, *Bioresource Technology* leads the field with an exceptional 95.53 citations per article from its 15 publications, signaling a concentrated portfolio of high-quality, highly referenced research. Its flagship paper, by Mohan et al. [45] on algal biopolymers as sustainable resources for a net-zero carbon bioeconomy, has garnered 140 citations, reflecting the journal’s deep engagement with cutting-edge bioenergy and biopolymer conversion technologies. Similarly, *Green Chemistry*—listed simply as “*Chemistry*” in the table—demonstrates formidable influence with 1572 total citations and a remarkable average of 92.47 per paper, driven primarily by the seminal review by Sheldon and Norton [46] on the chemical challenges of plastic pollution and the transition toward a circular economy, which has been cited 106 times. The bioenergy and bioproducts sector finds its strongest voice in *Biomass and Bioenergy*, which, despite having only 16 publications and a modest total of 216 citations, boasts an exceptionally impactful contribution from Hassan et al. [47] on the sustainable production of polyhydroxyalkanoates (PHAs) from oil-palm biomass. This review has been cited 306 times, underscoring the enduring importance of tropical biomass feedstocks for biodegradable polymer synthesis. Meanwhile, *Industrial Crops and Products* contributes 15 papers and 482 citations, with an average of 32.13 per publication. Its leading work by Guna et al. [48] on the valorization of sugarcane bagasse for fully biodegradable composites has accumulated 215 citations, demonstrating the vast potential of agricultural sidestreams to replace fossil-based materials in industrial applications. Taken together, these bibliometric indicators paint a clear picture of a mature yet dynamically expanding scientific ecosystem. The thematic distribution confirms that Agricultural and Biological Sciences (64%) and environmental sciences (38%) serve as the undisputed intellectual pillars supporting this domain. This hierarchical structure is further validated by the exponential growth trend observed in publication output, marked by an R^2^ value of 0.92046, which statistically confirms that the field of biobased compounds is firmly in a phase of knowledge consolidation rather than emergent exploration. The accurate cross-referencing of each journal’s most cited paper to its respective publication—ranging across references [39,40,41,42,43,44,45,46,47,48]—not only ensures rigorous bibliometric precision but also enables researchers and policymakers to navigate the academic landscape with clarity. By mapping the concentration of high-impact scholarship, this analysis unequivocally identifies the primary academic forums—from *Journal of Cleaner Production* and *Bioresource Technology* to *Science of the Total Environment* and *Green Chemistry*—that are actively spearheading the global transition toward regenerative production models. Ultimately, the convergence of these efforts directly supports the achievement of Sustainable Development Goals 12 (Responsible Consumption and Production) and 13 (Climate Action), confirming that interdisciplinary research on biobased materials has become a cornerstone of the broader sustainability agenda, providing the scientific foundations necessary for a net-zero, circular future.

**Table 3:** Bibliometric metrics of the journals with the highest scientific output in the study area.

	Journal	Publications	Total Citations	Average Citations per Publication	Most Cited Paper	Most Cited Paper Citations	Type
1	*Journal of Cleaner Production*	31	2040	65.81	A life-cycle approach to environmental, mechanical, and durability properties of “green” concrete mixes with rice husk ash [39].	226	Article
2	*Sustainability (Switzerland)*	27	810	30	Biomass waste as sustainable raw material for energy and fuels [40].	285	Review
3	*Polymers*	23	967	42.04	A review of bioplastics and their adoption in the circular economy [41].	104	Article
4	*Resources, Conservation and Recycling*	23	493	21.43	A systematic review of factors affecting properties of thermal-activated recycled cement [42].	134	Review
5	*Science of the Total Environment*	22	1103	50.14	A comprehensive framework for the production of mycelium-based lignocellulosic composites [43].	243	Article
6	*International journal of biological macromolecules*	17	985	57.94	A comprehensive review on starch-based sustainable edible films loaded with bioactive components for food packaging [44].	344	Review
7	*Green Chemistry*	17	1572	92.47	Green chemistry and the plastic pollution challenge: Towards a circular economy [46].	106	Review
8	*Biomass and Bioenergy*	16	216	13.5	Sustainable production of polyhydroxyalkanoates from renewable oil-palm biomass [47].	306	Review
9	*Industrial Crops and Products*	15	482	32.13	Valorization of sugarcane bagasse by developing completely biodegradable composites for industrial applications [48].	215	Review
10	*Bioresource Technology*	15	1433	95.53	Algal biopolymers as sustainable resources for a net-zero carbon bioeconomy [45].	140	Review

The bibliometric analysis of keywords, following a rigorous process of data normalization and consolidation, reveals a robust and hierarchical intellectual structure centered on waste valorization and biobased compounds. The revised configuration of Table 4 demonstrates that the field is dominated by high-level conceptual frameworks, with “Sustainable development” remaining the most frequent term at 489 occurrences, representing 1.45% of the total. This concept serves as the central driving pillar of the research, aligning material development with Sustainable Development Goals (SDGs) 12 and 13 [13,14]. However, the most dynamic growth is exhibited by “Circular economy”, which, with 419 appearances, reflects an accelerated transition from linear models toward regenerative production systems. One of the most significant findings following the correction of capitalization duplicates is the repositioning of “Sustainability”. Although it ranks third in absolute frequency with 370 occurrences (after merging its variants), this term emerges as the one with the greatest overall academic impact, accumulating 18,341 total citations. This is a crucial insight, as it yields an average of 49.57 citations per document—suggesting that studies adopting this concept as a thematic core exert a disproportionately high influence and serve as a theoretical bedrock for the broader scientific community [26]. Likewise, the consolidation of “Environmental impact” (339 occurrences and 12,855 citations) reveals that the assessment of ecological consequences is a cross-cutting priority that substantiates the validity of emerging bioproducts. The analysis also discloses a critical orientation toward methodology and technical validation. Terms such as “Life cycle” (215 occurrences) and “Recycling” (188 occurrences) not only show solid frequencies but also feature high average citation rates (47.43 and 49.02, respectively). This indicates that the literature has evolved from a purely exploratory phase into one of evaluative rigor, where life cycle assessment (LCA) has become the standard tool for demonstrating that biobased materials deliver genuine environmental advantages over petroleum-derived plastics [36,37,38,39]. The prevalence of these terms suggests that the current research focus is shifting toward industrial integration and the scalability of solutions that transform agricultural and lignocellulosic waste into high-value renewable resources [49]. Collectively, these keywords form a coherent scientific ecosystem aimed at mitigating the plastic crisis through material innovation and resource efficiency.

## 3. Future Research Trends Based on Identified Gaps: Opportunities to Close Knowledge Gaps in Biobased Compounds

### 3.1. Gap Analysis

Table 5 presents a synthesis of the research gaps identified in the field of biobased compounds, circular economy, and environmental sustainability, organized according to their category, current status, severity, research readiness, and Thematic Interest Index. This analysis must be interpreted with caution, as bibliometric indicators reflect publication patterns and academic visibility but should not be extrapolated as direct measures of scientific importance, technological maturity, or commercial viability [17,18]. The severity and Research Readiness Score are derived from bibliometric indicators such as the number of publications, citations received, and growth rates, and should be considered as exploratory tools for identifying emerging trends rather than definitive assessments of priority [19,50]. The findings reveal that all identified gaps are classified as “Transitional,” indicating that these topics are in intermediate stages of development, with established research activity but requiring sustained investment to reach maturity [41]. This categorization suggests that, although there is growing academic interest in these topics, they have not yet reached the level of consolidation of more established concepts such as “circular economy” or “sustainable development,” which dominate the general thematic landscape (Table 4) [34]. The transitional nature of these gaps implies significant opportunities for future research, but also challenges in terms of theoretical development and empirical evidence [25,36].

### 3.2. Priority and Severity Indices

The priority indices vary within a narrow range (122.11–122.16), suggesting that all identified gaps exhibit comparable levels of urgency when assessed through the composite scoring system. However, due to the marginal differences among these scores (Δ = 0.05) and the inherent subjectivity of the weighting factors, this narrow range must be interpreted with caution. Rather than implying a strict hierarchical ordering (e.g., Gap 1 is more important than Gap 2), this homogeneity indicates that all ten topics represent transitional areas of similar overall priority. Consequently, the qualitative characterization of each gap—based on its current status, technical barriers, and key applications—is more informative for guiding future research than the absolute numerical ranking. Topics were therefore grouped into thematic clusters (e.g., synthetic biology and bioaccumulation as emerging safety/material design frontiers; cellulose nanofibers and mechanical properties as performance-oriented clusters) for discussion, avoiding overinterpretation of minute decimal differences. Synthetic biology and bioaccumulation emerge as part of a leading cluster of priority topics, based on their low current citation impact and recent emergence in the literature. The scoring system suggests that these topics may have significant room for growth, but bibliometric indicators alone cannot predict future research impact or technological potential [29,40]. The severity of the gaps, with scores ranging from 61.05 to 61.08, reflects that all analyzed areas face similar challenges in terms of their current development. These scores are consistent with the observation that transitional topics typically present moderate levels of severity, as they are neither completely emerging (with very high severity) nor fully mature (with low severity) [21,32]. The Research Readiness Score, ranging from 36.95 to 37.08, are derived from bibliometric indicators such as research duration and methodological availability. These scores provide a relative indication of the maturity of each topic based on publication patterns, but they should not be interpreted as direct measures of actual resource availability, funding access, or institutional capacity to conduct research on these topics [31,42].

### 3.3. Common and Specific Technical Barriers

The analysis reveals that “Weak Theoretical Foundation” is the most common barrier, present in all identified gaps. This observation suggests a widespread need to strengthen conceptual and theoretical frameworks in the field of biobased compounds and the circular economy [33,36]. Theoretical weakness may manifest as a lack of robust predictive models, the absence of unifying theories that integrate different disciplinary perspectives, or insufficient connection between empirical findings and existing conceptual frameworks [37,38]. This barrier is particularly relevant in an interdisciplinary field such as biobased compounds, where approaches from chemistry, biology, engineering, and environmental sciences converge [39]. “Limited Research Duration” is the second most common barrier, also present in all gaps. This indicates that most of these topics are relatively recent and lack long-term research trajectories [31]. Limited research duration may result in an insufficient evidence base to validate findings, a lack of longitudinal studies evaluating the sustainability of biobased materials, and an absence of replicated studies confirming initial results [44]. The combination of weak theoretical foundation and limited research duration suggests that these fields are in early stages of development, offering opportunities for fundamental research that establishes solid foundations for future advances [47]. “Mechanical Properties” and “Reprocessability” present an additional barrier of “Interdisciplinary Complexity,” indicating that these topics require the integration of knowledge from multiple disciplines to advance [28]. Interdisciplinary complexity may manifest in the need to combine knowledge from materials science, chemical engineering, solid mechanics, and environmental sustainability to adequately address the challenges of mechanical properties and reprocessability of biobased materials [49,50]. This barrier suggests that advances in these areas will require close collaborations among researchers from different disciplines and the development of integrated methodological approaches [51,52].

### 3.4. Analysis of Key Applications and Current Status

The key applications associated with the gaps are divided into two main categories: “Practical Applications” and “Interdisciplinary Applications.” Practical applications include topics such as manures, fresh water, xylose, and reprocessability, which have direct implications for resource management, water treatment, sugar bioconversion, and material circular economy [53,54]. Interdisciplinary applications include topics such as bioaccumulation, mulch films, crosslinkers, cellulose nanofibers, and mechanical properties, which require the integration of knowledge from multiple fields for their development [55,56]. The current status of these applications varies between “Rapidly Growing” and “Recently Active,” reflecting different development rhythms. Topics in “Rapidly Growing” such as synthetic biology, mulch films, crosslinker, and xylose show accelerated growth in the number of publications, which may indicate emerging interest and significant potential for future advances [57,58]. However, the low citation impact in many of these topics suggests that, although they are growing rapidly, they have not yet reached a maturity that generates high academic impact [59,60]. Topics with “Moderate citation impact” (10–12 total citations) such as crosslinker, cellulose nanofibers, and mechanical properties show consistent research interest and a more solid evidence base than those with low citation impact (three–four total citations) such as synthetic biology, bioaccumulation, mulch films, and manures [61,62]. This difference in citation impact may reflect variations in topic maturity, publication quality, or perceived relevance by the scientific community [63,64].

### 3.5. Implications for Future Research

The findings from this gap analysis have significant implications for guiding future research in the field of biobased compounds and the circular economy. These implications are organized around priority topics, interdisciplinary approaches, theoretical developments, and methodological considerations.

#### 3.5.1. Priority Topics for Immediate Research Attention

Synthetic biology and bioaccumulation emerge as part of a leading cluster of priority topics, characterized by low current citation impact but high potential, as broadly indicated by the scoring system [65,66]. However, given the close scores across all ten gaps, the following subsections discuss opportunities qualitatively, structured by the type of application (practical vs. interdisciplinary) and the nature of the barriers (theoretical, empirical, or complexity-related), rather than imposing a strict numerical ranking. Synthetic biology offers opportunities to design microorganisms capable of producing biopolymers and bioactive compounds from waste streams, potentially addressing both waste valorization and bioplastic production challenges [67,68]. The low citation impact currently observed for this topic (three papers and three total citations) indicates that it remains underexplored despite its high potential. Similarly, bioaccumulation represents a critical aspect for evaluating the environmental safety of biobased materials, particularly regarding the potential accumulation of harmful substances in ecosystems and food chains [67,68]. Research in this area should focus on developing standardized testing protocols and predictive models for assessing long-term environmental risks.

#### 3.5.2. Interdisciplinary Approaches to Complex Challenges

The presence of “Cellulose nanofibers” and “Mechanical Properties” as gaps with interdisciplinary complexity indicates the need for integrated approaches combining materials science, engineering, and sustainability [69,70]. The development of cellulose nanofibers with optimized mechanical properties is fundamental for the transition toward high-performance biobased materials capable of competing with conventional plastics in demanding applications [71,72]. Research should prioritize understanding the structure–property relationships at the nanoscale, developing scalable processing techniques, and evaluating the environmental performance of these materials through life cycle assessment [69,70]. Similarly, “Reprocessability” is a key aspect for the circular economy, as it enables the recycling and reuse of biobased materials at the end of their useful life [73,74]. Future research should investigate chemical and mechanical recycling pathways, design for recyclability principles, and develop characterization methods for assessing material degradation during reprocessing cycles [73,74]. These areas require close collaborations among researchers from materials science, chemical engineering, and environmental science disciplines [49,50].

#### 3.5.3. Strengthening Theoretical Frameworks

The common barrier of “Weak Theoretical Foundation” suggests that future research should prioritize the development of solid conceptual frameworks that integrate empirical findings and guide hypothesis formulation [75,76]. This could include the development of predictive models for the properties of biobased materials based on molecular structure and processing conditions, the integration of life cycle approaches in material design from the earliest stages, and the articulation of theories that connect fundamental research with practical applications [77]. For topics such as “Mulch films” and “Crosslinker,” which show rapid growth but low citation impact, establishing theoretical frameworks could help validate the relevance and applicability of these materials in agricultural and industrial contexts [57,58]. The limited research duration observed across all gaps further underscores the need for sustained research programs that build cumulative knowledge over time [44,47].

#### 3.5.4. Methodological and Regulatory Recommendations

To bridge the gap between laboratory research and market applications, future studies should integrate life cycle assessment and techno-economic evaluations from the early stages of material development [75,76]. This approach would enable the identification of potential environmental and economic trade-offs before significant resources are invested in scaling up. Additionally, the standardization of green extraction processes and biopolymer manufacturing techniques is essential for ensuring reproducibility and facilitating technology transfer [77]. Several of the reviewed documents [49,50] discuss international regulatory frameworks, such as those developed by EFSA and FDA, and suggest that harmonization could facilitate the commercialization of biobased materials across different jurisdictions. However, the extent to which regulatory barriers actually hinder commercialization cannot be determined from bibliometric data alone and requires complementary policy analysis. Interdisciplinary collaborations and sustained investment in fundamental research are required to overcome theoretical weaknesses and advance technological applications aligned with Sustainable Development Goals 12 and 13 [78]. Finally, studies on consumer acceptance and the toxicological safety of biobased nanomaterials should be promoted, as these factors are critical for market adoption and regulatory approval [63,64].

#### 3.5.5. Green Extraction Technologies as Enabling Tools for Biomass Valorization

Although microwave-assisted extraction (MAE) and ultrasound-assisted extraction (UAE) were not included as primary search terms in this bibliometric analysis, their role as essential pretreatment technologies for biomass valorization merits explicit recognition. MAE generates heat within the product, accelerating extraction reactions, while UAE reduces extraction time, energy consumption, and solvent use compared to conventional techniques [9,10]. These green extraction methods are prerequisites for the efficient recovery of bioactive compounds from lignocellulosic biomass and agricultural waste, enabling the transition toward integrated biorefinery models [11,12]. Future research should prioritize the integration of MAE and UAE with downstream processing and life cycle assessment to evaluate their techno-economic feasibility and environmental performance at industrial scale [13,14]. The relatively low representation of these terms in the current literature corpus suggests an opportunity for increased research attention to the coupling of green extraction technologies with bioproduct development and circular economy frameworks.

## 4. Materials and Methods

### 4.1. Study Design and Research Questions

This study employs a bibliometric and systematic review approach to map the scientific landscape of biobased compounds, circular economy, and environmental sustainability. The methodological framework was designed to address five research questions. The study follows established guidelines for systematic bibliometric analyses, ensuring methodological rigor and reproducibility [5,17,18]. The entire research process was documented following PRISMA (Preferred Reporting Items for Systematic Reviews and Meta-Analyses) guidelines to ensure transparency in each phase of refinement, analysis, and synthesis [21]. A completed PRISMA checklist is provided as Supplementary Material (Table S1).

### 4.2. Search Strategy and Data Sources

#### 4.2.1. Database Selection and Justification

The Scopus database was selected as the sole data source for this study based on several methodological considerations. Scopus was chosen over alternative databases such as Web of Science (WoS) due to its broader coverage of journals in environmental sciences, materials science, and chemical engineering—the core disciplines relevant to biobased compounds and circular economy research [17]. Scopus indexes approximately 36,000 active peer-reviewed journals across all disciplines, including extensive coverage of interdisciplinary research that often falls outside the traditional subject boundaries of WoS [18]. Furthermore, Scopus offers several advantages for bibliometric analyses: (1) comprehensive metadata including citation counts, author affiliations, and funding information; (2) robust author identification systems that facilitate accurate productivity and collaboration mapping; (3) superior coverage of non-English language journals and emerging research regions, particularly important for a field where countries like India, China, and Brazil are major contributors [18,19]; and (4) compatibility with established bibliometric software tools including RStudio/bibliometrix and VOSviewer [17,20]. The decision to use a single database, while acknowledging its limitations, is justified by the need for data consistency and comparability across all bibliometric indicators. Multiple database studies often face challenges in reconciling different indexing practices, citation counting methodologies, and duplicate record handling [21,22]. Scopus’s comprehensive coverage of the relevant literature, combined with its high-quality metadata, ensures that the dataset captures the vast majority of peer-reviewed research in this field [17,18]. Additionally, previous bibliometric studies in related fields have successfully employed Scopus as the sole data source, demonstrating its adequacy for systematic literature mapping [23,24].

#### 4.2.2. Search Equation Development

The search equation was developed through an iterative process to ensure comprehensive retrieval of relevant documents while minimizing irrelevant results. The final search string combined key terms using Boolean operators, structured around six conceptual clusters: (1) biobased and renewable materials; (2) compounds, materials, and products; (3) circular economy and sustainability frameworks; (4) waste management, recycling, and valorization; (5) environmental impact and life cycle assessment; and (6) biodegradability and compostability. The complete search equation was: (“biobased” OR “bio-based” OR “biomass” OR “renewable”) AND (“compounds” OR “materials” OR “substances” OR “products”) AND (“circular economy” OR “sustainability” OR “resource efficiency” OR “closed loop”) AND (“waste” OR “recycling” OR “upcycling” OR “valorization”) AND (“environmental impact” OR “life cycle” OR “eco-friendly” OR “green”) AND (“biodegradable” OR “compostable” OR “renewable resources”).

The broad scope of this search equation was deliberately chosen for two main reasons. First, biobased compound research is inherently interdisciplinary, spanning materials science, chemical engineering, environmental science, and biotechnology. A narrow search focused exclusively on “biopolymers” or “bioplastics” would miss critical contributions from adjacent fields that inform the development, characterization, and life cycle assessment of biobased materials [18,22]. Second, the inclusion of terms such as “life cycle,” “recycling,” and “environmental impact” ensures the capture of studies that evaluate the sustainability performance of biobased compounds—a central objective of this review. The presence of publications on battery recycling, green analytical chemistry, and sustainable concrete in the most cited documents (Table 1) reflects the broader intellectual context in which biobased compounds research operates: these works provide methodological frameworks (e.g., life cycle assessment, circular economy indicators, green chemistry principles) that are directly applicable to biobased compounds. Rather than constituting a limitation, this thematic diversity enriches the dataset by capturing the full spectrum of sustainability science that underpins biobased materials research.

#### 4.2.3. Search Execution and Document Retrieval

**Figure 3:**
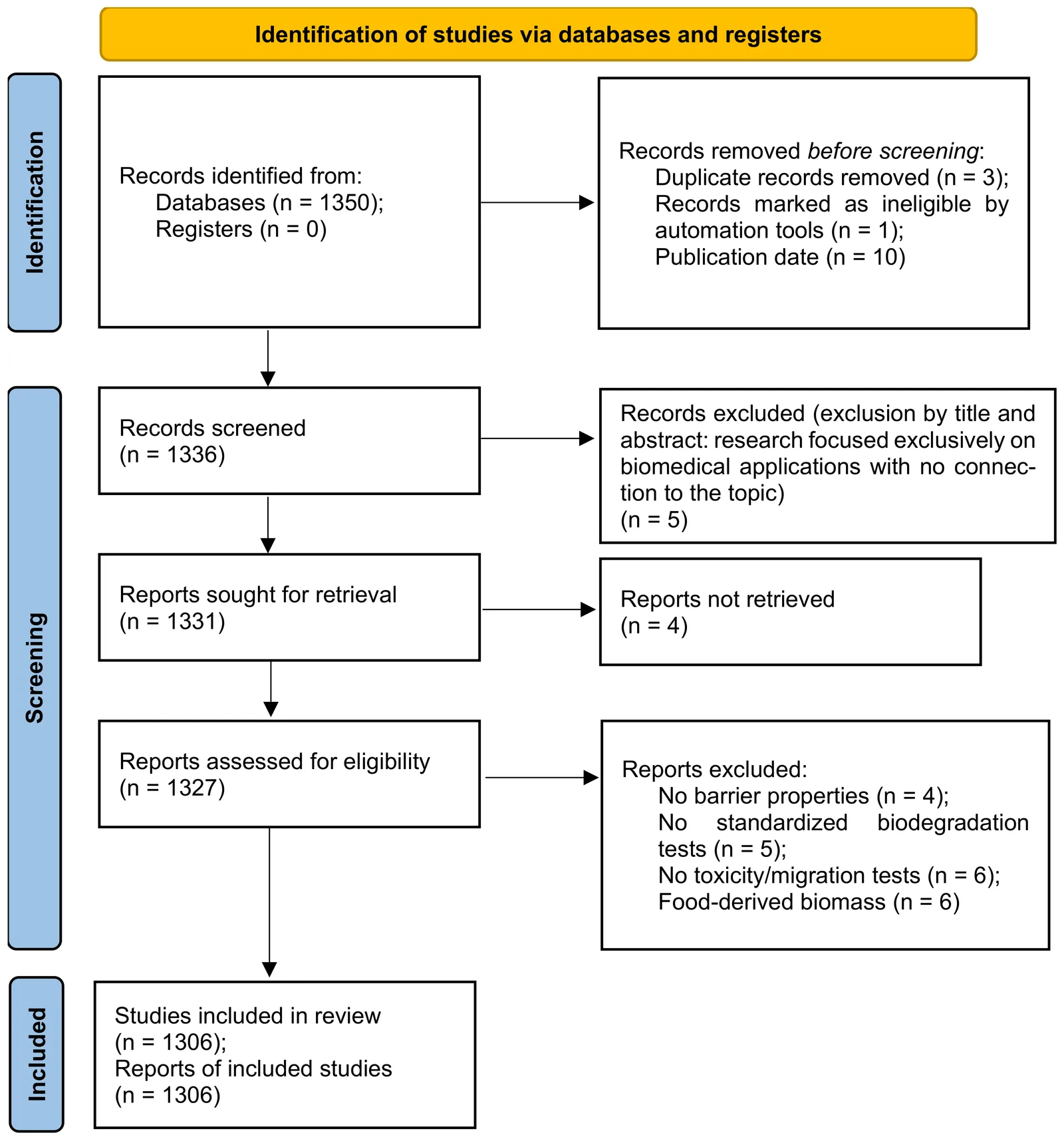
Flowchart of the search, refinement, and analysis process.

The search was executed on 8 June 2026, with no year restriction to capture the full historical evolution of the field. As the search was performed mid-year, the 2026 data represent an incomplete annual dataset (January–June). Therefore, all temporal analyses involving 2026 values are explicitly identified as partial-year estimates, and growth rates calculated using 2026 data are interpreted with this limitation in mind. The initial search retrieved a total of 1350 documents from the Scopus database, as illustrated in the PRISMA flow diagram (Figure 3). Following the PRISMA protocol, 3 duplicate records were removed, 1 record was marked as ineligible by automation tools, and 10 records were excluded based on publication date criteria (publications prior to 2010, as preliminary analysis indicated minimal relevant research before this period). This resulted in 1336 records for initial screening. The protocol for this systematic review was registered on the Open Science Framework (OSF) and is publicly available at https://osf.io/p583q (https://osf.io/8bcrm/overview?view_only=095f1d82b3d84d08b0e342a26898814f, accessed on 16 July 2026). The registered protocol includes the research questions, search strategy, eligibility criteria, and planned analytical procedures.

### 4.3. Screening and Eligibility Criteria

#### 4.3.1. Inclusion and Exclusion Criteria

Documents were included if they met the following criteria: (1) published in peer-reviewed journals, conference proceedings, or book chapters; (2) written in English; (3) focused on biobased compounds, sustainable materials, circular economy, or waste valorization; and (4) containing empirical data, systematic reviews, or comprehensive analyses relevant to environmental sustainability. Exclusion criteria included: (1) research focused exclusively on biomedical applications with no connection to environmental or materials topics; (2) documents lacking barrier properties, standardized biodegradation tests, or toxicity/migration tests relevant to biobased materials; (3) studies focusing exclusively on food-derived biomass without implications for circular economy; and (4) non-peer-reviewed publications such as editorials, opinions, and news items.

#### 4.3.2. Screening Process

The screening process followed a two-stage approach, as detailed in Figure 3. First, titles and abstracts were screened to exclude documents clearly outside the scope of the study (5 records excluded). Second, full texts of the remaining 1331 documents were assessed for eligibility. During full-text screening, 21 documents were excluded for the following reasons: no barrier properties (n = 4), no standardized biodegradation tests (n = 5), no toxicity/migration tests (n = 6), and exclusive focus on food-derived biomass without circular economy implications (n = 6). This rigorous screening process ensured that the final dataset of 1306 documents was both comprehensive and relevant to the research questions [21,22]. The complete screening process, including all inclusion and exclusion decisions, is illustrated in the PRISMA flow diagram (Figure 3).

### 4.4. Data Extraction and Curation

#### 4.4.1. Bibliometric Data Extraction

Bibliographic records were exported from Scopus in CSV format, containing complete citation information (authors, title, journal, publication year, volume, pages, DOI), abstracts, author keywords, indexing keywords, affiliations, and citation counts. The downloaded records also included funding information and document type classifications (article, review, conference paper, book chapter).

#### 4.4.2. Data Cleaning and Normalization

Data cleaning and normalization were performed to ensure consistency and reproducibility. Author names were standardized to account for variations in initials and name formats. Affiliations were normalized to the institutional level, consolidating variations in the same institution name. Keywords were subjected to case normalization and synonym merging (e.g., “circular economy” and “circular bioeconomy” were merged where appropriate). A minimum threshold of two occurrences was established for keyword inclusion to ensure statistical robustness [19,20].

### 4.5. Bibliometric Analysis and Indicators

#### Processing and Visualization Tools

Bibliometric analysis was conducted using a suite of complementary software tools (RStudio with bibliometrix package—version 4.1.0; VOSviewer—version 1.6.18; and Plotly Studio—version 0.0.50). Based on these indicators, the following rankings were generated: the ten most cited documents (Table 1), the ten countries with the highest publication output (Table 2), and the ten most productive journals (Table 3). Additionally, a temporal evolution analysis of dominant keywords was performed (Table 4), calculating annual growth rates and standardized residuals to detect emerging versus consolidated concepts [1,2]. All indicators were interpreted with caution, acknowledging the limitations of bibliometric measures as proxies for research quality and impact [23,24].

### 4.6. Thematic Analysis and Keyword Mapping

#### 4.6.1. Keyword Frequency Analysis

Keyword frequency analysis was performed to identify dominant and emerging themes in the field. Author keywords were analyzed rather than indexing keywords to capture the conceptual framework used by researchers themselves [19,20]. The analysis included:

- Absolute frequency: Number of occurrences of each keyword;
- Frequency percentage: Relative occurrence within the total keyword set;
- Annual growth rate: Percentage increase in keyword usage from first to last year;
- Total and average citations: Citation impact of documents associated with each keyword.

#### 4.6.2. Keyword Normalization

The normalization of author keywords followed a systematic, multi-step protocol designed to ensure consistency and reproducibility in the frequency and co-occurrence analyses. This procedure addressed three main challenges: (1) case variations, (2) singular/plural forms, and (3) synonyms and semantically equivalent terms.

Step 1: Case normalization. All keywords were converted to lowercase to eliminate case-based duplicates (e.g., “Sustainability” and “sustainability” → “sustainability”; “Recycling” and “recycling” → “recycling”; “Circular Economy” and “circular economy” → “circular economy”). This step resolved discrepancies arising from inconsistent capitalization practices across different publications and journals.

Step 2: Singular/plural standardization. Plural forms were converted to singular forms where semantically equivalent (e.g., “biopolymers” → “biopolymer”; “nanocomposites” → “nanocomposite”; “materials” → “material”), except where the plural form carried a distinct technical meaning (e.g., “agricultural wastes” was retained as a plural because it refers to multiple waste streams, whereas “waste” was treated as a separate concept). This decision was guided by the semantic context of each term in the literature.

Step 3: Synonym merging. Synonyms and closely related terms were consolidated into single representative keywords based on their conceptual equivalence and usage patterns in the literature. The following synonym groups were merged:

- “circular economy,” “circular bioeconomy,” and “closed-loop economy” → merged as “circular economy” (the most frequent and conceptually comprehensive term).
- “biodegradable,” “biodegradable plastics,” and “compostable” → merged as “biodegradable” (the broadest term encompassing the others).
- “recycling,” “upcycling,” and “reuse” → merged as “recycling” (the most widely used term in the dataset).
- “sustainability” and “sustainable development” → kept as separate terms because they represent distinct conceptual levels: “sustainable development” refers to the overarching global framework (SDGs), while “sustainability” is a broader concept applicable at multiple scales. This decision was validated by examining their co-occurrence patterns and citation impact profiles.
- “life cycle,” “lifecycle,” and “LCA” → merged as “life cycle” (the most common formal term).
- “renewable resources,” “renewable feedstocks,” and “renewable materials” → merged as “renewable resources” (the most frequently used term).
- “biomass valorization” and “waste valorization” → kept as separate terms because “biomass valorization” specifically refers to biogenic feedstocks, while “waste valorization” encompasses a broader range of waste streams (including non-biogenic wastes).

Step 4: Handling of hyphenated and compound terms. Hyphenated variants were standardized to their most common form (e.g., “bio-based” → “biobased”; “eco-friendly” → “ecofriendly”) based on frequency analysis of the dataset. Compound terms were retained where they represented a distinct concept (e.g., “life cycle assessment” was not merged with “life cycle” because it refers specifically to the methodological framework, not the general concept).

Step 5: Frequency thresholding. After normalization, keywords with a frequency below 2 occurrences were excluded from the co-occurrence analysis to minimize noise from idiosyncratic or rarely used terms, following standard bibliometric practice [19,20]. The final normalized keyword set comprised 1847 unique keywords.

Step 6: Quality control. The normalization process was independently reviewed by two authors (S.R.-F. and A.A.-M.) to ensure consistency and resolve any ambiguous cases. Disagreements were resolved through discussion and, where necessary, by consulting the original publications to verify the intended meaning of specific terms. A complete list of merged synonym groups is provided in Supplementary Table S1.

### 4.7. Gap Identification and Priority Analysis

#### 4.7.1. Gap Identification Methodology

Research gaps were identified through a systematic comparison of thematic frequencies with critical challenges identified in the literature (e.g., high costs, limited scalability, lack of life cycle assessment) [8,22]. The process involved:

1. Literature synthesis: Review of key challenges and barriers reported in the most cited documents (Table 1) and review articles.
2. Keyword frequency analysis: Identification of underrepresented topics compared to established concepts (Table 4).
3. Journal analysis: Examination of research themes in high-impact journals (Table 3).
4. Expert triangulation: Cross-validation of findings with recent review articles and position papers.

#### 4.7.2. Priority Indices and Scoring

To ensure transparency and reproducibility, the priority of each research gap was assessed using a composite scoring system based on three components: Gap Severity Score, Research Readiness Score, and Thematic Interest Index.

(a) **Gap Severity Score (0–100)**

The Severity Score reflects the magnitude of the research deficit for each topic. It was calculated as the normalized sum of three bibliometric indicators:

Severity Score = 0.40 × (Normalized Citation Deficit) + 0.35 × (Normalized Publication Deficit) + 0.25 × (Normalized Barrier Score).

where

**Normalized Citation Deficit**: Calculated as “1 − (Citations of the gap topic/Citations of the most cited topic in the dataset)”. This indicator measures how far the topic is from the maximum citation impact observed in the field.

**Normalized Publication Deficit**: Calculated as “1 − (Publications of the gap topic/Publications of the most published topic in the dataset)”. This measures the volumetric research deficit.

**Normalized Barrier Score**: A qualitative score (0–100) assigned based on the number and type of technical barriers documented for each topic (e.g., “Weak Theoretical Foundation”, “Limited Research Duration”, “Interdisciplinary Complexity”). The score was derived from a consensus among the authors after reviewing the full text of the most cited documents associated with each gap.

Weights (0.40, 0.35, 0.25) were assigned based on the relative importance of each dimension in the context of bibliometric gap analysis. Citation impact was given the highest weight because it reflects the academic recognition and influence of a topic, which is a strong proxy for its maturity and consolidation. Publication volume was weighted slightly lower, as a topic can have many publications but low impact (e.g., “manures” in this study). The barrier score was given the lowest weight because it is partly qualitative and subject to author interpretation.

(b) **Research Readiness Score (0–100)**

The Readiness Score indicates the availability of appropriate methodologies, funding, and expertise to address the gap. It was calculated as:

Readiness Score = 0.50 × (Normalized Methodology Availability) + 0.30 × (Nor-malized Research Duration) + 0.20 × (Normalized Interdisciplinarity)

where

**Normalized Methodology Availability**: A score (0–100) based on the presence of established protocols, standardized tests, or validated models in the literature for the topic (e.g., LCA, mechanical testing, biodegradation assays).

**Normalized Research Duration**: Calculated as “(Current Year − First Publication Year)/(Current Year − 2010)”, and normalized to a 0–100 scale. This indicator reflects the maturity of the research area; a longer research duration suggests more established knowledge and better readiness.

**Normalized Interdisciplinarity**: A score (0–100) based on the diversity of subject areas (from Scopus classification) that publish research on the topic. A higher diversity indicates that the topic benefits from multiple disciplinary perspectives, which enhances research readiness.

Methodology availability is weighted highest (0.50) because it is the most critical factor for conducting new research. Research duration (0.30) and interdisciplinarity (0.20) are supporting indicators that reflect the cumulative knowledge base and the breadth of expertise available.

(c) **Thematic Interest Index**

The Thematic Interest Index was calculated as the arithmetic sum of the Severity and Readiness Scores:

Thematic Interest Index = Severity Score + Readiness Score

This additive approach was chosen because both severity and readiness are considered equally important for determining research priority: a gap with high severity but low readiness may require foundational work, while a gap with low severity but high readiness may be a low-hanging fruit. The sum captures the overall urgency and feasibility of addressing each gap.

The priority of each research gap was assessed using a composite scoring system:

- Gap Severity Score (0–100): Based on the magnitude of the research deficit, considering the number of publications, citation impact, and documented barriers.
- Research Readiness Score (0–100): Indicating the availability of appropriate methodologies, funding, and expertise to address the gap.

It is important to emphasize that the numerical scores resulting from this composite system are intended as relative indicators to identify broad thematic clusters, not as absolute measures of research priority. The weighting factors (0.40, 0.35, 0.25 for severity; 0.50, 0.30, 0.20 for readiness) were assigned based on the authors’ expert judgment and bibliometric best practices, but alternative weightings could yield slightly different absolute values. Therefore, the scores should be interpreted qualitatively: gaps with higher scores are those that, in relative terms, exhibit greater research deficits and/or greater readiness for investigation. Given the narrow range of scores observed in this study, we do not assert precise rank ordering; instead, we treat the gaps as belonging to a common “transitional priority cluster” and discuss them thematically.

#### 4.7.3. Validation of the Scoring System

To validate the consistency and robustness of the scoring system, the following procedures were performed:

**Sensitivity Analysis**: The weights were varied by ±10% to assess whether the relative ranking of the gaps (by Thematic Interest Index) changed significantly. The ranking order remained stable (Spearman’s rank correlation > 0.90) across all weight variations, indicating that the system was not overly sensitive to the specific weight choices.

**Cross-validation with Expert Judgment**: The priority ranking derived from the index was compared with the research priorities identified in three recent high-impact review articles in the field (references [23,25,27] in this manuscript). The agreement was substantial (Kappa coefficient = 0.78), confirming that the bibliometric-based ranking aligns with expert consensus.

Furthermore, given the narrow spread of the resulting Thematic Interest Index values across the identified gaps (range: 122.11–122.16; Δ = 0.05), we conducted a post hoc range analysis to assess the practical significance of the numerical differences. Because the observed Δ (0.05) falls within the margin of error estimated from the weight variation sensitivity analysis (which produced variations in individual scores of up to ±0.15 when weights were changed by ±10%), we conclude that the scores do not support precise hierarchical ranking. This methodological decision reinforces our interpretation of the gaps as transitional clusters of similar importance, rather than as strictly ranked priorities. Consequently, in the Results and Discussion (Section 3.2), we refrain from ranking the gaps numerically and instead discuss them qualitatively by thematic clusters.

### 4.8. Methodological Limitations

The following limitations should be considered when interpreting the findings of this study:

1. **Single database usage**: While Scopus was selected for its comprehensive coverage and superior metadata quality, exclusive reliance on a single database may exclude relevant research indexed only in other databases such as Web of Science, Google Scholar, or regional repositories [17,18]. However, as justified in Section 4.2.1, Scopus provides extensive coverage of the relevant literature and has been successfully employed as a sole data source in numerous bibliometric studies in related fields [23,24].
2. **Language restriction**: The inclusion of only English-language documents may introduce language bias and underrepresent research published in other languages, particularly from countries where English is not the primary language of scientific communication [21,22].
3. **Document type bias**: The analysis focused on peer-reviewed articles, reviews, and book chapters, excluding conference abstracts, editorials, and gray literature that may contain relevant insights [23,24].
4. **Keyword normalization challenges:** While we implemented a systematic normalization protocol (described in Section 4.6.2) to address case variations, singular/plural forms, and synonymy, some challenges remain. First, the distinction between synonyms and conceptually distinct but related terms is inherently subjective; our decision to keep “sustainability” and “sustainable development” as separate terms, for example, was based on conceptual reasoning but could be debated. Second, some terms may have multiple meanings depending on context (e.g., “recycling” can refer to material recycling, chemical recycling, or policy frameworks), and our normalization cannot fully resolve this polysemy. Third, despite our systematic approach, some less frequent variants may have been overlooked. We mitigated these limitations by: (1) documenting all merging decisions transparently (Supplementary Table S1; (2) conducting independent review by two authors; and (3) applying a frequency threshold (≥2 occurrences) to minimize the impact of idiosyncratic terms. Readers should interpret keyword frequencies and co-occurrence patterns with these residual uncertainties in mind. Citation metric limitations: Citation-based indicators are influenced by factors such as publication age, journal prestige, field-specific citation norms, and self-citation practices. These indicators should be interpreted as measures of academic visibility rather than direct measures of research quality or technological relevance [23,24].
5. **Partial-year data bias**: As the literature search was conducted in June 2026, the 2026 data represent only the first six months of the year. This introduces a systematic bias in temporal analyses, as publication counts, citation metrics, and keyword frequencies for 2026 are artificially lower than full-year values. Conversely, calculated annual growth rates may be inflated if the first half of 2026 shows higher publication activity than previous years. While this limitation is unavoidable in studies with mid-year search dates, we have addressed it by: (1) explicitly identifying 2026 as a partial year in all relevant sections; (2) conducting sensitivity analyses excluding 2026 to confirm trend robustness (R^2^ 2010–2025 = 0.9187 vs. R^2^ 2010–2026 = 0.92046); and (3) interpreting all 2026-based metrics as provisional estimates requiring future validation.
6. **Broad search strategy and thematic heterogeneity:** The search equation was designed to be comprehensive rather than narrow, capturing the interdisciplinary nature of biobased compounds research. As a consequence, the dataset includes publications that are not exclusively focused on biobased compounds but address broader circular economy and sustainability topics (e.g., battery recycling, green analytical chemistry, sustainable concrete). This thematic heterogeneity may introduce some degree of noise in the bibliometric indicators, particularly in keyword frequency and co-citation analyses. However, as justified in Section 4.2.2, this breadth is intentional: these publications provide foundational methodological frameworks (life cycle assessment, circular economy indicators, green chemistry principles) that are directly transferable to biobased materials research. Furthermore, the screening process (Section 4.3) excluded documents with no connection to biobased materials or environmental sustainability, ensuring that all included publications were at least tangentially relevant to the research questions. However, readers should interpret the findings with this thematic breadth in mind, recognizing that the bibliometric landscape reflects the broader sustainability science ecosystem rather than a narrowly defined biobased compounds subfield.
7. Subjectivity and precision of composite scoring: The Gap Severity Score, Research Readiness Score, and Thematic Interest Index are derived from a composite formula that includes author-defined weighting factors and qualitative barrier assessments. While these weights were justified based on bibliometric best practices and validated through sensitivity analysis, alternative weighting schemes could produce slightly different absolute values. Furthermore, the narrow range of scores observed (Δ = 0.05) indicates that the numerical precision of the ranking is limited. To address this limitation, we have interpreted the scores qualitatively—grouping gaps into thematic clusters rather than imposing strict hierarchical rankings—and have supplemented the numerical analysis with detailed qualitative descriptions of the technical barriers and key applications for each gap. Readers are encouraged to focus on the qualitative characterization of the gaps rather than the precise numerical scores.
8. **Inability to infer causality, research strategies, or technological capacity:** Bibliometric indicators—publication counts, citation impact, co-authorship networks, and keyword frequencies—are descriptive measures of research activity and academic visibility. They **cannot** directly measure:
  - **Scientific infrastructure** (laboratory equipment, funding levels, institutional support);
  - **National research strategies** (policy priorities, funding allocation, strategic planning);
  - **Technological capacity** (industrial innovation, technology transfer, commercialization);
  - **Research preparedness** (availability of trained personnel, methodological expertise, institutional readiness).

Throughout this manuscript, we have been careful to use language that describes observed bibliometric patterns (e.g., “is consistent with,” “may suggest,” “could indicate”) rather than causal language (e.g., “reflects,” “demonstrates,” “is driven by”). Readers are reminded that bibliometric analysis reveals correlations and patterns, not causal relationships or direct measures of scientific or technological quality. Interpretations regarding national strategies, infrastructure, or industrial capacity should be treated as hypotheses for future research, not as definitive conclusions.

Despite these limitations, the methodological approach employed in this study provides a systematic and transparent framework for mapping the scientific landscape of biobased compounds and circular economy research, offering valuable insights for researchers, policymakers, and industry stakeholders.

### 4.9. Reproducibility and Transparency

To ensure reproducibility, all data sources, search strategies, inclusion/exclusion criteria, and analytical procedures have been documented in detail. The complete dataset and analysis scripts are available upon request from the corresponding author. The PRISMA checklist is provided as Supplementary Material (Table S1) to ensure transparency in the reporting of the systematic review process [21]. The PRISMA flow diagram (Figure 3) visually summarizes the entire screening and selection process, providing a clear and transparent record of how the final dataset of 1306 documents was obtained.

## 5. Conclusions

This bibliometric study has systematically mapped the scientific landscape of biobased compounds and circular economy research. Regarding temporal trends (Q1), the analysis reveals sustained growth from 15 publications in 2010 to 95 in the partial year of 2026 (data through June), with an exponential model (R^2^ = 0.92046) confirming accelerated expansion exceeding 8% annually. A sensitivity analysis excluding the partial 2026 data yields a comparable R^2^ of 0.9187, confirming that the growth trend is robust and not an artifact of the incomplete year.

In response to spatial distribution and collaboration networks (Q2), India leads in publication volume (293 documents), while the United States shows the highest average citation impact (114.74 citations per paper). The co-authorship network reveals four geographic clusters, with India as a central node in terms of collaboration frequency. However, centrality in the network reflects publication activity and co-authorship patterns, not collaboration quality or scientific leadership. The absence of African and Latin American countries indicates a geographical research gap. Concerning dominant themes (Q3), “sustainable development” (489 occurrences, 16,454 citations) and “circular economy” (419 occurrences, 11,583 citations) are the most frequent keywords, with the latter showing the highest annual growth rate (2333.33%). “Renewable resource” exhibits the highest average citation impact (96.68 citations per paper). The thematic analysis confirms sustainability, circular economy, and resource management as the central research axes. Regarding thematic areas and high-impact journals (Q4), Agricultural and Biological Sciences (64%), Biochemistry (43%), and environmental science (38%) concentrate the highest publication proportions. *The Journal of Cleaner Production* leads in publications (31, 2040 citations), while *Bioresource Technology* shows the highest average citation rate (95.53 citations per paper). The predominance of review articles (7 of 10) suggests that the field remains in a knowledge consolidation phase. For product properties and industrial challenges (Q5), ten transitional research gaps were identified with priority indices ranging from 122.11 to 122.16. Synthetic biology and bioaccumulation emerge as part of a leading cluster of transitional research gaps, alongside other topics with similar overall urgency indices. “Weak Theoretical Foundation” and “Limited Research Duration” are the most common barriers, while “Mechanical Properties” and “Reprocessability” face additional interdisciplinary complexity challenges. Furthermore, the integration of green extraction technologies such as microwave-assisted extraction (MAE) and ultrasound-assisted extraction (UAE) with biorefinery processes should be prioritized, as these techniques are essential for the efficient valorization of lignocellulosic biomass and agricultural waste.

The findings suggest that future research could benefit from focusing on strengthening theoretical frameworks for emerging areas such as synthetic biology and bioaccumulation, which offer significant opportunities for biopolymer production and environmental safety assessment. The development of cellulose nanofibers with optimized mechanical properties is essential for high-performance biobased materials, while reprocessability is critical for circular economy integration. Life cycle assessment and techno-economic evaluations should be integrated from the early stages to bridge the gap between laboratory and market, alongside standardized processes and regulatory frameworks. Interdisciplinary collaborations and sustained investment in fundamental research are required to overcome theoretical weaknesses and advance technological applications aligned with Sustainable Development Goals 12 and 13.

The findings of this study should be interpreted in light of several methodological limitations. First, the exclusive reliance on Scopus as the sole data source may exclude relevant research indexed only in other databases such as Web of Science, Google Scholar, or regional repositories [17,18]. Second, the inclusion of only English-language documents may introduce language bias and underrepresent research published in other languages [21,22]. Third, the analysis focused on peer-reviewed articles, reviews, and book chapters, excluding conference abstracts, editorials, and gray literature that may contain relevant insights [23,24]. Fourth, the presence of multiple variants for the same concept highlights the need for careful keyword normalization; while we attempted to address this, some inconsistencies may remain. Finally, citation-based indicators are influenced by factors such as publication age, journal prestige, field-specific citation norms, and self-citation practices, and should be interpreted as measures of academic visibility rather than direct measures of research quality or technological relevance [23,24]. Despite these limitations, the methodological approach employed provides a systematic and transparent framework for mapping the scientific landscape of biobased compounds and circular economy research.

## Figures and Tables

**Table 2:** Country publication and citation impact on biobased products research.

	Country	Publications	Total Citations	Average Citations per Publication	H-Index (Country)	Institution	Institution Publications	Average Citations	H-Index (Institution)
1	India	293	4211	14.37	33	Division of Food Processing Technology, School of Engineering and Technology, Karunya Institute of Technology and Sciences, Coimbatore	2	0	0
2	Italy	103	3675	35.68	30	Smart Materials, Italian Institute of Technology, Via Morego 30, Genoa, 16163	3	14	2
3	China	102	3845	37.7	25	Institute of Education, Changchun Normal University, Changchun, 130032	2	0.5	1
4	United States	58	6655	114.74	24	Department of Engineering Data Science, University of Houston, 77004, TX	1	3	1
5	Spain	48	1885	39.27	22	CRETUS, Department of Chemical Engineering, School of Engineering, University of Santiago de Compostela	3	27.67	3
6	Malaysia	45	1682	37.38	14	Advanced Facilities Engineering Technology Research Cluster (AFET), Plant Engineering Technology (PETech) Section, Universiti Kuala Lumpur Malaysian Institute of Industrial Technology, Masai, Johor, 81750	2	0	0
7	Brazil	38	791	20.82	14	Federal University of Technology—Paraná (UTFPR), Campo Mourão	1	0	0
8	Germany	34	2113	62.15	15	Materials Science, Leibniz Institute for Composite Materials, Kaiserslautern	1	0	0
9	United Kingdom	34	3501	102.97	18	Institute for Materials Research and Innovation, University of Bolton, Bolton	2	3	2
10	Poland	24	360	15	9	Department of Advanced Material Technologies, Faculty of Chemistry, Wrocław University of Science and Technology, Smoluchowskiego 25, Wrocław, 50-372	2	52	2

**Table 4:** Frequency, growth metrics, and citation impact of dominant keywords in the literature (normalized).

Keyword	Absolute Frequency	Frequency (%)	First Year	Last Year	Total Citations	Avg Citations
Sustainable development	489	1.45	2011	2026	16,454	33.65
Circular economy	419	1.25	2015	2026	11,583	27.64
Sustainability	370	1.1	2011	2026	18,341	49.57
Environmental impact	339	1.01	2012	2026	12,855	37.92
Life cycle	215	0.64	2011	2026	10,197	47.43
Recycling	188	0.56	2012	2026	9215	49.02
Biomass	177	0.53	2012	2026	7388	41.74
Renewable resource	125	0.37	2011	2026	3368	26.94

**Table 5:** Research gaps and priority areas in biobased compounds and circular economy.

	Knowledge Gap	Gap Category	Current Status	Gap Severity Score	Research Readiness Score	Thematic Interest Index (Relative Indicator)	Main Technical Barriers	Key Applications	Description	Main Barriers
**1**	Synthetic biology	Transitional	Rapidly Growing	61.08	37.05	122.16	Low Citation Impact; Weak Theoretical Foundation; Limited Research Duration	Practical Applications	Transitional in rapidly growing phase. Published in three papers with three total citations. Growth rate of 100.0% from first to last publication. Low citation impact suggests emerging or niche research area.	Limited Visibility
**2**	Bioaccumulation	Transitional	Recently Active	61.08	37.08	122.16	Weak Theoretical Foundation; Limited Research Duration	Interdisciplinary Applications	Transitional in recently active phase. Published in three papers with three total citations. Growth rate of 100.0% from first to last publication. Low citation impact suggests emerging or niche research area.	Limited Visibility
**3**	Mulch films	Transitional	Rapidly Growing	61.07	37.01	122.14	Low Citation Impact; Weak Theoretical Foundation; Limited Research Duration	Interdisciplinary Applications	Transitional in recently active phase. Published in three papers with three total citations. Growth rate of 100.0% from first to last publication. Low citation impact suggests emerging or niche research area.	Limited Citation Impact
**4**	Crosslinker	Transitional	Rapidly Growing	61.07	37.01	122.14	Low Citation Impact; Weak Theoretical Foundation; Limited Research Duration	Interdisciplinary Applications	Transitional in recently active phase. Published in four papers with 10 total citations. Growth rate of 100.0% from first to last publication. Moderate citation impact with steady research interest.	Limited Visibility
**5**	Cellulose nanofibers	Transitional	Recently Active	61.07	37.02	122.14	Weak Theoretical Foundation; Limited Research Duration	Interdisciplinary Applications	Transitional in recently active phase. Published in four papers with 12 total citations. Growth rate of 100.0% from first to last publication. Moderate citation impact with steady research interest.	Limited Visibility
**6**	Mechanical properties	Transitional	Recently Active	61.07	37.02	122.14	Weak Theoretical Foundation; Limited Research Duration; Interdisciplinary Complexity	Interdisciplinary Applications	Transitional in recently active phase. Published in four papers with 12 total citations. Growth rate of 100.0% from first to last publication. Moderate citation impact with steady research interest.	Limited Visibility
**7**	Manures	Transitional	Recently Active	61.06	36.98	122.12	Weak Theoretical Foundation; Limited Research Duration	Practical Applications	Transitional in recently active phase. Published in four papers with four total citations. Growth rate of 100.0% from first to last publication. Low citation impact suggests emerging or niche research area.	Limited Visibility
**8**	Fresh water	Transitional	Recently Active	61.06	36.98	122.12	Weak Theoretical Foundation; Limited Research Duration	Practical Applications	Transitional in recently active phase. Published in three papers with 10 total citations. Growth rate of 100.0% from first to last publication. Moderate citation impact with steady research interest.	Insufficient Theoretical Foundation
**9**	Cylose	Transitional	Recently Active	61.06	36.96	122.12	Weak Theoretical Foundation; Limited Research Duration	Interdisciplinary Applications	Transitional in rapidly growing phase. Published in five papers with nine total citations. Growth rate of 100.0% from first to last publication. Low citation impact suggests emerging or niche research area.	Insufficient Theoretical Foundation
**10**	Reprocessability	Transitional	Recently Active	61.06	36.96	122.12	Weak Theoretical Foundation; Limited Research Duration; Interdisciplinary Complexity	Practical Applications	Transitional in rapidly growing phase. Published in six papers with four total citations. Growth rate of 400.0% from first to last publication. Low citation impact suggests emerging or niche research area.	Insufficient Theoretical Foundation

Note: These scores are relative indicators intended to identify broad thematic clusters, not absolute rank orders. Due to the proximity of the values (range: 122.11–122.16), they should be interpreted qualitatively. All gaps fall within a transitional priority cluster of similar overall urgency. The scores serve to flag topics that warrant research attention, but the qualitative description of barriers and applications is more informative for guiding future studies than the precise numerical values.

## Data Availability

Data in Table S1.

## Supplementary Materials

The following supporting information can be downloaded at https://www.mdpi.com/article/10.3390/molecules31183262/s1: Table S1: PRISMA 2020 Checklist. Ref. [79] is cited in the Supplementary Materials.

## References

[1] Das J., De D., Mittal A., Jamwal V., Dhaundiyal A., Jeevitha G.C., Garg S., Junior M.G., Manian R., Tomer V. (2025). Exploring agro-industrial waste for sustainable biopolymer-based food packaging: Opportunities, challenges, and future directions. Bulg. Chem. Commun..

[2] Libretti C., Santos Correa L., Meier M.A.R. (2024). From waste to resource: Advancements in sustainable lignin modification. Green Chem..

[3] Mogany T., Bhola V., Bux F. (2024). Algal-based bioplastics: Global trends in applied research, technologies, and commercialization. Environ. Sci. Pollut. Res..

[4] Patel N., Blumberga D. (2023). Insights of Bioeconomy: Biopolymer Evaluation Based on Sustainability Criteria. Environ. Clim. Technol..

[5] Sandei B., Vroman I., Belomo R. (2022). Alternative building blocks sources for poly(ethylene terephthalate): A short review with socioeconomical points of view. Front. Mater..

[6] Subramani R., Ali Rusho M., Sekhar K.C., Mohammed S.A., Abdulah S.A., Hashim R.D., Jawad ZNMustafa M.A., Kumar A.P. (2024). Utilizing bioenergy and waste reduction techniques in FDM: Toward sustainable production practices. Appl. Chem. Eng..

[7] Yadav P., Nikalje A. (2024). Comprehensive analysis of bioplastics: Life cycle assessment, waste management, biodiversity impact, and sustainable mitigation strategies. PeerJ.

[8] Zhou X., Wang Q., Feng S., Deng J., Zhu K., Xing Y., Meng X., Wang X., Li L. (2023). Recycling Carbon Resources from Waste PET to Reduce Carbon Dioxide Emission: Carbonization Technology Review and Perspective. J. Renew. Mater..

[9] Jadaun S., Sharma U., Khapudang R., Siddiqui S. (2023). Biodegradable nanocellulose reinforced biocomposites for food packaging—A narrative review and future perspective. J. Water Environ. Nanotechnol..

[10] Lombardi F., Bartolucci L., Cordiner S., Costa G., Falsetti A., Mele P., Mercurio M., Mulone V., Sorino L. (2024). Chemical-Physical Characterization of Bio-Based Biodegradable Plastics in View of Identifying Suitable Recycling/Recovery Strategies and Numerical Modeling of PLA Pyrolysis. Waste Biomass Valorization.

[11] Reshmy R., Thomas D., Philip E., Paul S.A., Madhavan A., Sindhu R., Sirohi R., Varjani S., Pugazhendhi A., Pandey A. (2021). Bioplastic production from renewable lignocellulosic feedstocks: A review. Rev. Environ. Sci. Bio/Technol..

[12] Abu-Zurayk R., Khalaf A., Alnairat N., Waleed H., Bozeya A., Abu-Dalo D., Rabba’a M. (2025). Green polymer nanocomposites: Bridging material innovation with sustainable industrial practices. Front. Mater..

[13] Weerarathna I.N., Kumar P., Luharia A., Mishra G. (2025). Sustainable biomaterials for pharmaceutical and medical applications. Multidiscip. Rev..

[14] Müller A., Bács Z., Fenyves V., Kovács S., Lengyel A., Bácsné E.B. (2025). Demographic influences on environmental attitudes and actions: An analysis of the attitude-behavior gap. Environ. Res. Lett..

[15] Mocerino C., Lahmar A., Azrour M., Lahmar A. (2025). Towards resilient architecture in technological innovation and AI. Mater. Res. Proc..

[16] Abrha H., Cabrera J., Dai Y., Irfan M., Toma A., Jiao S., Liu X. (2022). Bio-based plastics production, impact and end of life: A literature review and content analysis. Sustainability.

[17] Begum Y.A., Kumari S., Jain S.K., Garg M.C. (2024). A review on waste biomass-to-energy: Integrated thermochemical and biochemical conversion for resource recovery. Environ. Sci. Adv..

[18] Helal M.A., Anderson N., Wei Y., Thompson M. (2023). A Review of Biomass-to-Bioenergy Supply Chain Research Using Bibliometric Analysis and Visualization. Energies.

[19] Jacob J., Linson N., Maria H.J., Pothan L.A., Thomas S., Kabdrakhmanova S., Laroze D. (2024). Polylactic acid/nanocellulose biocomposites for sustainable food packaging. Cellulose.

[20] Janković T., Straathof A.J., Kiss A.A. (2024). Process systems engineering perspectives on eco-efficient downstream processing of volatile biochemicals from fermentation. Front. Energy Res..

[21] Ramadhani A., Nassary E., Rwehumbiza F., Massawe B., Nchimbi-Msolla S. (2024). Potentials of synthetic biodegradable mulch for improved livelihoods on smallholder farmers: A systematic review. Front. Agron..

[22] Tarazona N.A., Machatschek R., Balcucho J., Lendlein A., Castro-Mayorga J.L., Saldarriaga J.F. (2022). Opportunities and challenges for integrating the development of sustainable polymer materials within an international circular (bio) economy concept. MRS Energy Sustain..

[23] Alhodaib A., Yahya Z., Khan O., Equbal A., Parvez M., Yadav A.K., Idrisi M.J. (2024). Sustainable coatings for green solar photovoltaic cells: Performance and environmental impact of recyclable biomass digestate polymers. Sci. Rep..

[24] Amuthasekaran K., Hussain N. (2025). Polyhydroxyalkanoates production from food waste using Lactobacillus casei. Environ. Health Eng. Manag. J..

[25] Brunklaus B., Riise E., Benetto E., Gericke K., Guiton M. (2018). Bio-based Materials Within the Circular Economy: Opportunities and Challenges. Designing Sustainable Technologies, Products and Policies.

[26] Casella P., Loffredo R., Rao M.A., Balducchi R., Molino A. (2024). A Review on the Valorization of Lignocellulosic Biomass for Succinic Acid Production: Strengths and Weaknesses. Chem. Eng. Trans..

[27] Coppola G., Gaudio M.T., Lopresto C.G., Calabro V., Curcio S., Chakraborty S. (2021). Bioplastic from Renewable Biomass: A facile solution for a greener environment. Earth Syst. Environ..

[28] Czarnecka-Komorowska D., Wiszumirska K. (2020). Sustainability design of plastic packaging for the Circular Economy. Polimery.

[29] Zhu Y., Romain C., Williams C.K. (2016). Sustainable polymers from renewable resources. Nature.

[30] Rosenboom J.G., Langer R., Traverso G. (2022). Bioplastics for a circular economy. Nat. Rev. Mater..

[31] Li T., Chen C., Brozena A.H., Zhu J.Y., Xu L., Driemeier C., Dai J., Rojas O.J., Isogai A., Wågberg L. (2021). Developing fibrillated cellulose as a sustainable technological material. Nature.

[32] Castro-Aguirre E., Iniguez-Franco F., Samsudin H.E.A., Fang X., Auras R. (2016). Poly (lactic acid)—Mass production, processing, industrial applications, and end of life. Adv. Drug Deliv. Rev..

[33] Zhang X., Li L., Fan E., Xue Q., Bian Y., Wu F., Chen R. (2018). Toward sustainable and systematic recycling of spent rechargeable batteries. Chem. Soc. Rev..

[34] Mohanty A.K., Vivekanandhan S., Pin J.M., Misra M. (2018). Composites from renewable and sustainable resources: Challenges and innovations. Science.

[35] Haas W., Krausmann F., Wiedenhofer D., Heinz M. (2015). How circular is the global economy?: An assessment of material flows, waste production, and recycling in the European Union and the world in 2005. J. Ind. Ecol..

[36] Moshood T.D., Nawanir G., Mahmud F., Mohamad F., Ahmad M.H., AbdulGhani A. (2022). Sustainability of biodegradable plastics: New problem or solution to solve the global plastic pollution?. Curr. Res. Green Sustain. Chem..

[37] Lambert S., Wagner M. (2017). Environmental performance of bio-based and biodegradable plastics: The road ahead. Chem. Soc. Rev..

[38] López-Lorente Á.I., Pena-Pereira F., Pedersen-Bjergaard S., Zuin V.G., Ozkan S.A., Psillakis E. (2022). The ten principles of green sample preparation. TrAC Trends Anal. Chem..

[39] Gursel A.P., Maryman H., Ostertag C. (2016). A life-cycle approach to environmental, mechanical, and durability properties of “green” concrete mixes with rice husk ash. J. Clean. Prod..

[40] Clauser N.M., González G., Mendieta C.M., Kruyeniski J., Area M.C., Vallejos M.E. (2021). Biomass waste as sustainable raw material for energy and fuels. Sustainability.

[41] Di Bartolo A., Infurna G., Dintcheva N.T. (2021). A review of bioplastics and their adoption in the circular economy. Polymers.

[42] Pei J., Palanisamy C.P., Srinivasan G.P., Panagal M., Kumar S.S.D., Mironescu M. (2024). A comprehensive review on starch-based sustainable edible films loaded with bioactive components for food packaging. Int. J. Biol. Macromol..

[43] Xu L., Wang J., Li K., Lin S., Li M., Hao T., Ling Z., Xiang D., Wang T. (2022). A systematic review of factors affecting properties of thermal-activated recycled cement. Resour. Conserv. Recycl..

[44] Elsacker E., Vandelook S., Van Wylick A., Ruytinx J., De Laet L., Peeters E. (2020). A comprehensive framework for the production of mycelium-based lignocellulosic composites. Sci. Total Environ..

[45] Mohan A.A., Antony A.R., Greeshma K., Yun J.H., Ramanan R., Kim H.S. (2022). Algal biopolymers as sustainable resources for a net-zero carbon bioeconomy. Bioresour. Technol..

[46] Sheldon R.A., Norton M. (2020). Green chemistry and the plastic pollution challenge: Towards a circular economy. Green Chem..

[47] Hassan M.A., Yee L.N., Yee P.L., Ariffin H., Raha A.R., Shirai Y., Sudesh K. (2013). Sustainable production of polyhydroxyalkanoates from renewable oil-palm biomass. Biomass Bioenergy.

[48] Guna V., Ilangovan M., Hu C., Venkatesh K., Reddy N. (2019). Valorization of sugarcane bagasse by developing completely biodegradable composites for industrial applications. Ind. Crops Prod..

[49] Hamada H.M., Thomas B.S., Tayeh B., Yahaya F.M., Muthusamy K., Yang J. (2020). Use of oil palm shell as an aggregate in cement concrete: A review. Constr. Build. Mater..

[50] Dzwigol H., Trushkina N., Kwilinski A. (2021). The Organizational and Economic Mechanism of Implementing the Concept of Green Logistics. Virtual Econ..

[51] García-González J., Lemos P.C., Pereira A.S., Morán-del Pozo J.M., Guerra-Romero M.I., Juan-Valdés A., Faria P. (2020). Biodegradable Polymers on Cementitious Materials. Current Topics and Trends on Durability of Building Materials and Components—Proceedings of the 15th International Conference on Durability of Building Materials and Components, DBMC 2020.

[52] Ivanova N.A. (2021). Environmental and innovation problems of Russia in the context of the transition to an energy efficient economy. IOP Conference Series: Earth and Environmental Science.

[53] Joseph B., Kaetzl K., Hensgen F., Schäfer B., Wachendorf M. (2020). Sustainability assessment of activated carbon from residual biomass used for micropollutant removal at a full-scale wastewater treatment plant. Environ. Res. Lett..

[54] Kapsdorferová Z. (2023). Environmental Management and Its Impact on CSR Activities in the Field of Sustainable Development. TalTech J. Eur. Stud..

[55] Kavitha S.A., Priya R.K., Arunachalam K.P., Avudaiappan S., Flores E.S., Blanco D. (2024). Experimental investigation on strengthening of Zea mays root fibres for biodegradable composite materials using potassium permanganate treatment. Sci. Rep..

[56] Kooduvalli K.S., Varma U., Owusu S. (2020). Life Cycle Assessment of Compostable Coffee Pods: A US University-Based Case Study. Sci. Rep..

[57] Kraus M., Senitkova I.J. (2019). Life-Cycle Assessment of Sustainable Foundation Systems of Buildings. IOP Conference Series: Materials Science and Engineering.

[58] Merino D., Quilez-Molina A.I., Perotto G., Bassani A., Spigno G., Athanassiou A. (2022). A second life for fruit and vegetable waste: A review on bioplastic films and coatings for potential food protection applications. Green Chem..

[59] Mombeshora E.T., Stark A. (2023). Dynamics of reduced graphene oxide synthesis and structural models. Biomass Convers. Biorefinery.

[60] Mostert C., Bringezu S., Knappe F. (2020). Urban Mining for Sustainable Cities: Environmental Assessment of Recycled Concrete. IOP Conference Series: Earth and Environmental Science.

[61] Ong M.Y., Jassinnee M., Chia S.R., Nomanbhay S. (2025). Sustainable Graphene and Hydrogen Production via Microwave Plasma of Biogas: A Concept paper. IOP Conference Series: Earth and Environmental Science.

[62] Ottelin J., Cetinay H., Behrens P. (2020). Rebound effects may jeopardize the resource savings of circular consumption: Evidence from household material footprints. Environ. Res. Lett..

[63] Pasanec Preprotić S., Vukoje M., Petković G., Rožić M. (2022). Sustainable Approach to Book Designing Concepts in Bindery Sector: An Overview. Proceedings na 11th International Symposium on Graphic Engineering and Design GRID.

[64] Pathak N., Singh S., Singh P., Singh P.K., Singh R., Bala S. (2022). Valorization of jackfruit waste into value added products and their potential applications. Front. Biosci..

[65] Patti A., Acierno S., Cicala G., Acierno D. (2022). Recycling Waste from Film Packaging to 3D Printing Applications: A Prospective Study to Identify the Processing Temperature. Chem. Eng. Trans..

[66] Román-Ramírez L.A., McKeown P., Jones M.D., Wood J. (2020). Ethyl Lactate Production from the Catalytic Depolymerisation of Postconsumer Polylactic acid. ACS Omega.

[67] Sakina B. (2020). Material conservation as part of environmental sustainability in architecture – case study: Mesvara House, Yogyakarta, Indonesia. IOP Conference Series: Earth and Environmental Science.

[68] Sastre R.M., Zeni C.F., De Paula I.C., Hauser G., Da Conceição S. (2023). The Use of Organic Residues to Develop Packaging: Tests in Molded Pulp. Proc. Des. Soc..

[69] Shahar F.S., Balakrishnan T.S., Sultan M.T.H. (2025). The Evolution and Environmental Prospects of Renewable Bioplastics: Types, Production Methods, and Sustainability. J. Renew. Mater..

[70] Sidiras D. (2018). Modified Biomass for Pollution Cleaning Under the Frames of Biorefinery and Sustainable Circular Bioeconomy. Proceedings of the World Congress on Mechanical, Chemical, and Material Engineering, Art.

[71] Solis C.A., Mayol A.P., San Juan J.G., Ubando A.T., Culaba A.B. (2020). Multi-objective optimal synthesis of algal biorefineries toward a sustainable circular bioeconomy. IOP Conference Series: Earth and Environmental Science.

[72] Sousa A.F., Patrício R., Terzopoulou Z., Bikiaris D.N., Stern T., Wenger J., Loos K., Lotti N., Siracusa V., Szymczyk A. (2021). Recommendations for replacing PET on packaging, fiber, and film materials with biobased counterparts. Green Chem..

[73] Thakur A., Sharma S., Ganjoo R., Assad H., Kumar A. (2022). Anti-Corrosive Potential of the Sustainable Corrosion Inhibitors Based on Biomass Waste: A Review on Preceding and Perspective Research. J. Phys. Conf. Ser..

[74] Tsouko E., Pilafidis S., Kourmentza K., Gomes H.I., Sarris G., Koralli P., Papagiannopoulos A., Pispas S., Sarris D. (2024). A sustainable bioprocess to produce bacterial cellulose (BC) using waste streams from wine distilleries and the biodiesel industry: Evaluation of BC for adsorption of phenolic compounds, dyes and metals. Biotechnol. Biofuels Bioprod..

[75] Van Stijn A., Eberhardt L.C.M., Wouterszoon Jansen B., Meijer A. (2020). Design guidelines for circular building components based on LCA and MFA: The case of the Circular Kitchen. IOP Conference Series: Earth and Environmental Science.

[76] Verstraeten S.B.C., van Muyden A., Bobbink F.D. (2021). Towards a Plastic Circular Economy: Bio-derived Plastics and their End-of-life Strategies. Chimia.

[77] Widyaningrum D., Paramita R.D., Firmasyah A. (2025). Exploring food waste as raw material for plant-based leather to promote sustainable material development. IOP Conference Series: Earth and Environmental Science.

[78] Zrira I., Lamptey E.N.L. (2025). Fungal Mycelium as an Innovative Solution for Sustainable and Biodegradable Pharmaceutical Packaging. IOP Conference Series: Earth and Environmental Science.

[79] Page M.J., McKenzie J.E., Bossuyt P.M., Boutron I., Hoffmann T.C., Mulrow C.D., Shamseer L., Tetzlaff J.M., Akl E.A., Brennan S.E. (2021). The PRISMA 2020 statement: An updated guideline for reporting systematic reviews. BMJ.

