# Supplementary material for: Biobased Compounds and the Circular Economy: A Bibliometric Review of Waste Valorization and Environmental Sustainability
Source: Molecules. 2026 Sep 15;31(18):3262. doi: 10.3390/molecules31183262 (PMC13609598; doi:10.3390/molecules31183262)
Supplement: Supplementary file 1 [file molecules-31-03262-s001.zip › molecules-4373222-Table S1.pdf]

PRISMA 2020 Checklist

Section and Topic	Item #	Checklist item	Location where item is reported
**TITLE**			
Title	1	Identify the report as a systematic review.	The title includes "Bibliometric Review", and the abstract explicitly defines it as a "comprehensive bibliometric and systematic review"
**ABSTRACT**			
Abstract	2	See the PRISMA 2020 for Abstracts checklist.	A detailed abstract is provided, covering the rationale (plastic crisis), methodology (Scopus search), results (exponential growth), and conclusions (research gaps)
**INTRODUCTION**			
Rationale	3	Describe the rationale for the review in the context of existing knowledge.	Section 1 details the global plastic crisis (367M tons), the low recycling rate (10%), and the massive underutilization of lignocellulosic biomass
Objectives	4	Provide an explicit statement of the objective(s) or question(s) the review addresses.	Sections 1 and 4.1 state the objective to map the knowledge structure and address five specific research questions (Q1–Q5) regarding trends, geography, themes, journals, and scalability
**METHODS**			
Eligibility criteria	5	Specify the inclusion and exclusion criteria for the review and how studies were grouped for the syntheses.	Section 4.3.1 lists inclusion (peer-reviewed, English, biobased focus) and exclusion criteria (biomedical only, lack of barrier properties,

Section and Topic	Item #	Checklist item	Location where item is reported
			food-derived biomass)
Information sources	6	Specify all databases, registers, websites, organisations, reference lists and other sources searched or consulted to identify studies. Specify the date when each source was last searched or consulted.	Section 4.2.1 and 4.2.3: Scopus was the sole source. The search was executed on June 08, 2026
Search strategy	7	Present the full search strategies for all databases, registers and websites, including any filters and limits used.	Section 4.2.2 provides the complete Boolean search string containing six conceptual clusters
Selection process	8	Specify the methods used to decide whether a study met the inclusion criteria of the review, including how many reviewers screened each record and each report retrieved, whether they worked independently, and if applicable, details of automation tools used in the process.	Section 4.3.2 describes a two-stage screening process (titles/abstracts then full text) following PRISMA guidelines
Data collection process	9	Specify the methods used to collect data from reports, including how many reviewers collected data from each report, whether they worked independently, any processes for obtaining or confirming data from study investigators, and if applicable, details of automation tools used in the process.	Section 4.4.1: Bibliographic records were exported from Scopus in CSV format with complete metadata
Data items	10a	List and define all outcomes for which data were sought. Specify whether all results that were compatible with each outcome domain in each study were sought (e.g. for all measures, time points, analyses), and if not, the methods used to decide which results to collect.	Section 3.1: Outcomes include temporal trends, geographical leadership, co-authorship networks, and high-impact themes
	10b	List and define all other variables for which data were sought (e.g. participant and intervention characteristics, funding sources). Describe any assumptions made about any missing or unclear information.	Section 4.4.1 and 4.5.1: Variables include author affiliations, funding info, and document types
Study risk of bias	11	Specify the methods used to assess risk of bias in the included studies, including details of the tool(s) used, how many reviewers	No formal bias tool for

Section and Topic	Item #	Checklist item	Location where item is reported
assessment		assessed each study and whether they worked independently, and if applicable, details of automation tools used in the process.	individual studies was used (typical for bibliometrics), but Section 4.5.1 notes that citation metrics are interpreted with caution as proxies for quality
Effect measures	12	Specify for each outcome the effect measure(s) (e.g. risk ratio, mean difference) used in the synthesis or presentation of results.	Measures include annual growth rate, R^2 coefficient (0.9654), H-index, and a composite Priority Index for research gaps
Synthesis methods	13a	Describe the processes used to decide which studies were eligible for each synthesis (e.g. tabulating the study intervention characteristics and comparing against the planned groups for each synthesis (item #5)).	Section 4.3: Eligibility was determined by technical criteria such as the presence of empirical data on biodegradation, barrier properties, and toxicity
	13b	Describe any methods required to prepare the data for presentation or synthesis, such as handling of missing summary statistics, or data conversions.	Section 4.4.2: Data was cleaned and normalized, including standardizing author names, institutional affiliations, and merging keyword synonyms
	13c	Describe any methods used to tabulate or visually display results of individual studies and syntheses.	Section 4.5.1: Used RStudio/bibliometrix for statistics, VOSviewer for network maps (co-authorship/keywords), and Plotly Studio for interactive graphs
	13d	Describe any methods used to synthesize results and provide a rationale for the choice(s). If meta-analysis was performed, describe the model(s), method(s) to identify the presence and extent of statistical heterogeneity, and software package(s) used.	Section 4.1: Employs scientometric and systematic mapping to address Q1–Q5, using RStudio to process the 1,306-

Section and Topic	Item #	Checklist item	Location where item is reported
			document dataset
	13e	Describe any methods used to explore possible causes of heterogeneity among study results (e.g. subgroup analysis, meta-regression).	Section 4.5.1: Explored through geographic and thematic clustering in VOSviewer to identify regional collaboration and emerging subdomains
	13f	Describe any sensitivity analyses conducted to assess robustness of the synthesized results.	Section 4.4.2 and 4.7.1: Applied a minimum threshold of two occurrences for keywords and used expert triangulation to validate research gaps
Reporting bias assessment	14	Describe any methods used to assess risk of bias due to missing results in a synthesis (arising from reporting biases).	Section 4.8 acknowledges limitations such as single database usage and language restriction (English only)
Certainty assessment	15	Describe any methods used to assess certainty (or confidence) in the body of evidence for an outcome.	Section 4.7.2 defines a composite scoring system (Gap Severity and Research Readiness) to assess the status of research gaps
**RESULTS**			
Study selection	16a	Describe the results of the search and selection process, from the number of records identified in the search to the number of studies included in the review, ideally using a flow diagram.	Section 4.2.3 and Figure 3: 1,350 records identified; 1,306 documents included after removing duplicates and ineligible records
	16b	Cite studies that might appear to meet the inclusion criteria, but which were excluded, and explain why they were excluded.	Section 4.4.2: 21 documents were excluded during full-text review for lacking barrier properties, standardized

PRISMA 2020 Checklist

Section and Topic	Item #	Checklist item	Location where item is reported
			biodegradation tests, or toxicity tests
Study characteristics	17	Cite each included study and present its characteristics.	Table 1 lists the top 10 most cited documents with journals, years, and document types
Risk of bias in studies	18	Present assessments of risk of bias for each included study.	Table 1 presents total citations and average citations per year for the leading papers
Results of individual studies	19	For all outcomes, present, for each study: (a) summary statistics for each group (where appropriate) and (b) an effect estimate and its precision (e.g. confidence/credible interval), ideally using structured tables or plots.	Table 1 presents total citations and average citations per year for the leading papers
Results of syntheses	20a	For each synthesis, briefly summarise the characteristics and risk of bias among contributing studies.	Section 2: Most high-impact papers are reviews (9 of 10), indicating a knowledge consolidation phase. Caution is noted regarding citation impact vs. quality
	20b	Present results of all statistical syntheses conducted. If meta-analysis was done, present for each the summary estimate and its precision (e.g. confidence/credible interval) and measures of statistical heterogeneity. If comparing groups, describe the direction of the effect.	Section 2: Reports exponential growth (Figure 1), country leadership (Table 2), and keyword frequencies like "Sustainable development" (489)
	20c	Present results of all investigations of possible causes of heterogeneity among study results.	Section 2: Identified four main collaboration clusters (e.g., India-Turkey, US-China-Canada) and thematic breakdowns by discipline
	20d	Present results of all sensitivity analyses conducted to assess the robustness of the synthesized results.	Section 4.1: Gap analysis validated 10 transitional areas (e.g., synthetic

Section and Topic	Item #	Checklist item	Location where item is reported
			biology) using the Priority Index and expert cross-validation
Reporting biases	21	Present assessments of risk of bias due to missing results (arising from reporting biases) for each synthesis assessed.	Section 4.8: The authors assess reporting biases by identifying three main risks: single database usage (Scopus), which may exclude relevant research from other repositories; language restriction (English only), which introduces a bias against research in other languages; and document type bias, as the study excludes grey literature and conference abstracts
Certainty of evidence	22	Present assessments of certainty (or confidence) in the body of evidence for each outcome assessed.	Sections 4.7.2 and 4.8: The certainty of the evidence regarding research gaps is evaluated through a composite scoring system (Severity and Readiness), though these are explicitly defined as exploratory tools rather than definitive assessments. Additionally, the authors note that citation-based indicators should be interpreted as measures of academic visibility rather than direct measures of research quality, indicating a

Section and Topic	Item #	Checklist item	Location where item is reported
			limitation in the certainty of performance metrics
**DISCUSSION**			
Discussion	23a	Provide a general interpretation of the results in the context of other evidence.	Discussion: Field has matured from feasibility to systemic integration; India leads in volume while the US/UK lead in visibility
	23b	Discuss any limitations of the evidence included in the review.	Section 2: Notes that production in biomass-rich countries can be qualitatively heterogeneous despite high volume
	23c	Discuss any limitations of the review processes used.	Section 4.8: Limitations include single database usage (Scopus), language restriction (English only), and keyword normalization challenges
	23d	Discuss implications of the results for practice, policy, and future research.	Section 5: Highlights the need for interdisciplinary collaboration, strengthening theoretical frameworks, and alignment with SDGs 12 and 13
**OTHER INFORMATION**			
Registration and protocol	24a	Provide registration information for the review, including register name and registration number, or state that the review was not registered.	The report does not provide an external registration name or number (e.g., PROSPERO). It specifies that the study follows the PRISMA guidelines

Section and Topic	Item #	Checklist item	Location where item is reported
			and that the research process was documented to ensure transparency
	24b	Indicate where the review protocol can be accessed, or state that a protocol was not prepared.	A standalone pre-published protocol is not mentioned. However, the study states that its methodological framework, including search strategies and eligibility criteria, is documented in detail within the Materials and Methods section. Additionally, a PRISMA checklist is provided as Supplementary Material (Table S1).
	24c	Describe and explain any amendments to information provided at registration or in the protocol.	Not applicable; there are no amendments described in the text, as no prior registration or external protocol is referenced
Support	25	Describe sources of financial or non-financial support for the review, and the role of the funders or sponsors in the review.	Funding: UCV, project PC-2025-002
Competing interests	26	Declare any competing interests of review authors.	The authors declare no conflicts of interest
Availability of data, code and other materials	27	Report which of the following are publicly available and where they can be found: template data collection forms; data extracted from included studies; data used for all analyses; analytic code; any other materials used in the review.	Section 4.9: The complete dataset and analysis scripts are available upon request from the corresponding author

From: Page MJ, McKenzie JE, Bossuyt PM, Boutron I, Hoffmann TC, Mulrow CD, et al. The PRISMA 2020 statement: an updated guideline for reporting systematic reviews. BMJ 2021;372:n71. doi: 10.1136/bmj.n71. This work is licensed under CC BY 4.0. To view a copy of this license, visit https://creativecommons.org/licenses/by/4.0/
